# An archaeal lid-containing feruloyl esterase degrades polyethylene terephthalate

**DOI:** 10.1038/s42004-023-00998-z

**Published:** 2023-09-11

**Authors:** Pablo Perez-Garcia, Jennifer Chow, Elisa Costanzi, Marno Gurschke, Jonas Dittrich, Robert F. Dierkes, Rebecka Molitor, Violetta Applegate, Golo Feuerriegel, Prince Tete, Dominik Danso, Stephan Thies, Julia Schumacher, Christopher Pfleger, Karl-Erich Jaeger, Holger Gohlke, Sander H. J. Smits, Ruth A. Schmitz, Wolfgang R. Streit

**Affiliations:** 1 Department of Microbiology and Biotechnology, University of Hamburg, Hamburg, Germany; 2 Institute for General Microbiology, Christian-Albrechts-Universität zu Kiel, Kiel, Germany; 3 Center for Structural Studies (CSS), Heinrich Heine University Düsseldorf, Düsseldorf, Germany; 4 Institute for Pharmaceutical and Medicinal Chemistry, Heinrich Heine University Düsseldorf, Düsseldorf, Germany; 5 Institute of Molecular Enzyme Technology (IMET), Heinrich Heine University Düsseldorf, Jülich, Germany; 6 Institute of Bio- and Geosciences IBG-1: Biotechnology, Forschungszentrum Jülich GmbH, Jülich, Germany; 7 Institute for Bio- and Geosciences (IBG-4: Bioinformatics), Forschungszentrum Jülich, Jülich, Germany; 8 Institute for Biochemistry, Heinrich Heine University Düsseldorf, Düsseldorf, Germany

**Keywords:** Biocatalysis, Hydrolases, Enzyme mechanisms, X-ray crystallography

## Abstract

Polyethylene terephthalate (PET) is a commodity polymer known to globally contaminate marine and terrestrial environments. Today, around 80 bacterial and fungal PET-active enzymes (PETases) are known, originating from four bacterial and two fungal phyla. In contrast, no archaeal enzyme had been identified to degrade PET. Here we report on the structural and biochemical characterization of PET46 (RLI42440.1), an archaeal promiscuous feruloyl esterase exhibiting degradation activity on semi-crystalline PET powder comparable to IsPETase and LCC (wildtypes), and higher activity on bis-, and mono-(2-hydroxyethyl) terephthalate (BHET and MHET). The enzyme, found by a sequence-based metagenome search, is derived from a non-cultivated, deep-sea *Candidatus* Bathyarchaeota archaeon. Biochemical characterization demonstrated that PET46 is a promiscuous, heat-adapted hydrolase. Its crystal structure was solved at a resolution of 1.71 Å. It shares the core alpha/beta-hydrolase fold with bacterial PETases, but contains a unique lid common in feruloyl esterases, which is involved in substrate binding. Thus, our study widens the currently known diversity of PET-hydrolyzing enzymes, by demonstrating PET depolymerization by a plant cell wall-degrading esterase.

## Abstract (web summary)

Microbial enzymes are capable of degrading certain synthetic polymers, with most polyethylene terephthalate (PET) degrading enzymes known to derive from bacteria or fungi. Here, the authors describe an archaeal originating feruloyl-esterase PET46 enzyme with a flexible lid domain and PET degradation capability.

## Introduction

The global use of synthetic and fossil fuel-derived polymers on a multi-million-ton scale for over eight decades and the lack of concepts for recycling have led to an unprecedented accumulation of plastics of diverse sizes and blends in all ecological niches including the deep-ocean^1–5^. Plastic litter serves as a carrier for many microorganisms that can attach to their surface, constituting the so-called plastisphere^6–8^. Many studies have described the microbial communities colonizing most commodity polymers such as polyethylene (PE), polypropylene (PP), or polystyrene (PS), but also polyethylene terephthalate (PET) or polyamides (PA), through 16 S rDNA amplicon or metagenomic sequencing, and less often by FISH or DGGE analysis^8–11^. Most studies focused exclusively on bacterial lineages, while only a few identified eukaryotes or archaea in addition (see Jacquin et al.^12^). While it has been speculated that some of these attached microorganisms might potentially be involved in the degradation of the polymers, it is more likely that most of them will simply use the plastics as a biocarrier or metabolize the additives but are not able to break down the polymers themselves^13,14^.

Nevertheless, in recent years, several studies have identified microbial enzymes that are able to degrade some of these synthetic polymers, including PET, polyurethane (PUR), PA, and a few others from mainly renewable sources^13,15^. To date, approximately 200 enzymes have been described to act on these polymers (PAZy database^16^), most of them being esterases, amidases, and oxygenases. Many of these proteins have low conversion rates, show promiscuous activity, or are only active on oligomers. Even though some Euryarchaeota (*e.g*., Thermoplasmatales) and TACK-archaea (*e.g*., Thaumarchaeota, Crenarchaeota) have been found to colonize plastic particles of various sizes^17,18^, no plastic-active enzyme of clear archaeal origin had been identified to break down a synthetic polymer^19,20^.

In the case of PET, most degrading enzymes derive from bacteria, including Actinomycetota^21–23^, Pseudomonadota^24–26^, Bacillota^27,28^, and recently Bacteroidota^29^ and Chloroflexota^30^. The best characterized bacterial enzymes include the IsPETase from *Ideonella sakaiensis*^26^ and the Leaf-branch Compost Cutinase^22^ (LCC). Few enzymes have been identified in fungi (Eukarya), including *Candida antarctica* CalB, *Humicola insolens* HiC, and *Fusarium solani* FsC^31^. These enzymes share some common features: They are cutinases or esterases, their catalytic triad comprises Ser-Asp-His, the active site is fairly exposed to the solvent, and they are deprived of any lid domain. Furthermore, aromatic (Trp, Phe, Tyr) and Met residues within the catalytic pocket contribute to the binding of PET and the formation of the oxyanion hole^13,32,33^.

Within this work, we describe and characterize PET46 (NCBI accession RLI42440.1), an enzyme from archaeal origin that hydrolyzes PET polymer. The enzyme is encoded in the metagenome-assembled genome (MAG) of the Candidatus Bathyarchaeota archaeon B1_G2, a member of the TACK group that was found at the Guaymas Basin^34^. The experimentally established crystal structure of the protein is similar to bacterial PET-degrading enzymes but reveals several unique features. These include differences in the amino acid composition in and around the active site compared to its bacterial and eukaryotic counterparts. Furthermore, the enzyme’s structure shows high homology to feruloyl esterases, and a flexible lid domain covers its active site, which is not a common trait for PETases. These findings add to the scaffold diversity of PET-active enzymes and strongly suggest that more enzymes could be able to degrade PET, which possibly have not yet been discovered by strict sequence and structural homology searches.

## Results

### Profile Hidden Markov Model (HMM) search identifies the archaeal PETase PET46

**Fig. 1:**
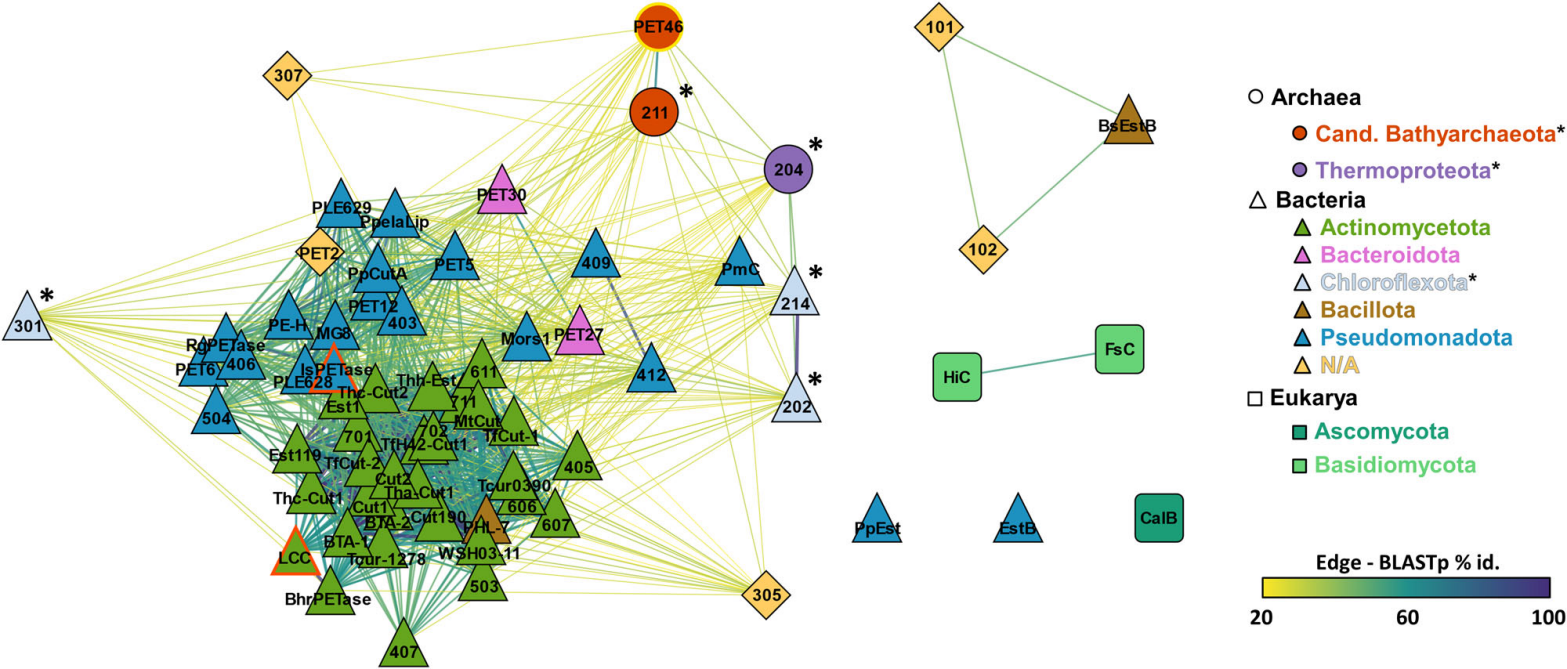
PET-degrading enzymes in Archaea, Bacteria and Eukarya. The amino acid sequence of PET46 was included in a sequence similarity network (SSN) analysis with other published PETases from Archaea (circles), Bacteria (triangles), and Eukarya (squares) collected in PAZy^16^. Edges connecting two nodes indicate a BLASTp e-value < 0.01, and color indicates the percentage identity. The enzymes PET46, IsPETase and LCC, compared in depth in this study, are highlighted. *Note: In the study by Erickson et al.^60^, the source organisms of enzymes 101–307 are not detailed and only defined as “environmental sample”. Taxonomic affiliation was derived from the best BLASTp hit from the NCBI nr-database if seq. id. > 95%. All-against-all BLASTp results are found in Supplementary Data 2.

Previous research identified PET esterases in bacteria and two fungal phyla (Fig. 1). Since hitherto no archaeal PETase had been described, we asked whether archaeal esterases might as well be capable to hydrolyze PET. To address this question, we performed global database searches using publicly available microbial genomes and metagenomes from NCBI’s non-redundant database using a previously published HMM-based search approach^25,35^.

We selected PET46 as a putative archaeal PET-degrading esterase (Fig. 1) based on bit score, sequence length, and initial 3D model analysis. Its sequence originates from a recently identified TACK archaeon found at a deep-sea marine sediment from the Guaymas Basin (Gulf of California, Mexico). The host strain Candidatus Bathyarchaeota archaeon B1_G2 has not yet been cultivated, but is part of a genome reconstruction project^34,36^. PET46 is encoded on a short 3,316 bp contig (QMYN01000264.1) by a 786 bp ORF coding for an alpha/beta-hydrolase (RLI42440.1) with 262 aa and a predicted molecular weight of 29.4 kDa (Supplementary Fig. 1). The other ORFs in the contig code for a tRNA-deacylase and two ribosomal proteins (Supplementary Fig. 1). PET46 showed only low sequence similarity to bacterial PETases^16^. The best hit with a BLASTp score of only 41.6 (query cov. 82%, seq. id. 26%) was the type I^37^ PETase Est1 from *Thermobifida alba* (Fig. 1).

### Amino acid sequence and structural analyses imply that PET46 is a feruloyl esterase

For an initial characterization, the PET46 amino acid sequence was subjected to a more detailed bioinformatics analysis. Thereby, we identified a predicted G-x-S-x-G motif which is a common trait of α/β serine hydrolases^38^. Amongst others, we identified conserved domains of dipeptidyl aminopeptidase/acylaminoacyl peptidase (DAP2), acetyl xylan esterase (AXE1), dienelactone hydrolase (DLH) and lysophospholipase (PldB, Supplementary Fig. 1). A BLASTp search against the non-redundant database results in 89 hits (query cov. > 80%, seq. id > 40%), from which only 24 are archaeal homologs, exclusively from the TACK-group. Most of them derive from Bathyarchaeota, and only three hits are related to the phylum Thermoproteota/Crenarchaeota. Interestingly, 65 homologs were found in Bacteria, especially in Firmicutes/Bacillota (Supplementary Fig. 1).

For further characterization, we produced PET46 wildtype (WT) heterologously in *E. coli* BL21(DE3) carrying an N-terminal 6xHis-tag (Supplementary Fig. 2). The recombinant and purified protein was used for crystallization and additional biochemical tests.

Crystals of PET46 were obtained by sitting-drop vapor diffusion after 3–4 weeks. The best PET46 crystal grew in space group P 6_1_ 2 2 and diffracted to a resolution of 1.71 Å (Table 1). We could unambiguously model the protein chain in the electron density between residues 1–269. The final model was refined to *R*_work_/*R*_free_ values of 15.23/17.27 and deposited to the PDB with accession ID 8B4U (Supplementary Data 1). All data collection and refinement statistics are reported in Table 1.

**Table 1:** Data collection and refinement statistics.

**PET46**	
*PDB ID*	8B4U
Data collection	
Wavelength [Å]	0.9793
Space group	P6_1_22
Unit Cell Parameters	
a, b, c [Å]	79.29 79.29 171.55
α, β, γ [°]	90.00 90.00 120.00
Resolution [Å]	68.67–1.71 (1.74–1.71)
Number of unique reflections	34,960 (1,813)
R_merge_	0.066 (1.370)
R_meas_	0.071 (1.457)
R_pim_	0.024 (0.487)
<I/σ(I)>	15.7 (1.5)
CC^1/2^	0.999 (0.559)
Completeness [%]	99.4 (99.9)
Multiplicity	8.5 (8.5)
Refinement	
Resolution [Å]	38.68–1.71
Number of reflections	34953
R_work_/R_free_ [%]	15.2 / 17.3
MolProbity overall score	1.24
R.M.S. deviations	
bond length [Å]	0.011
bond angles [°]	1.013
Ramachandran plot	
Favored [%]	98.88
Allowed [%]	1.12
Outliers [%]	0

Values in parenthesis refer to the highest resolution shell.

**Fig. 2:**
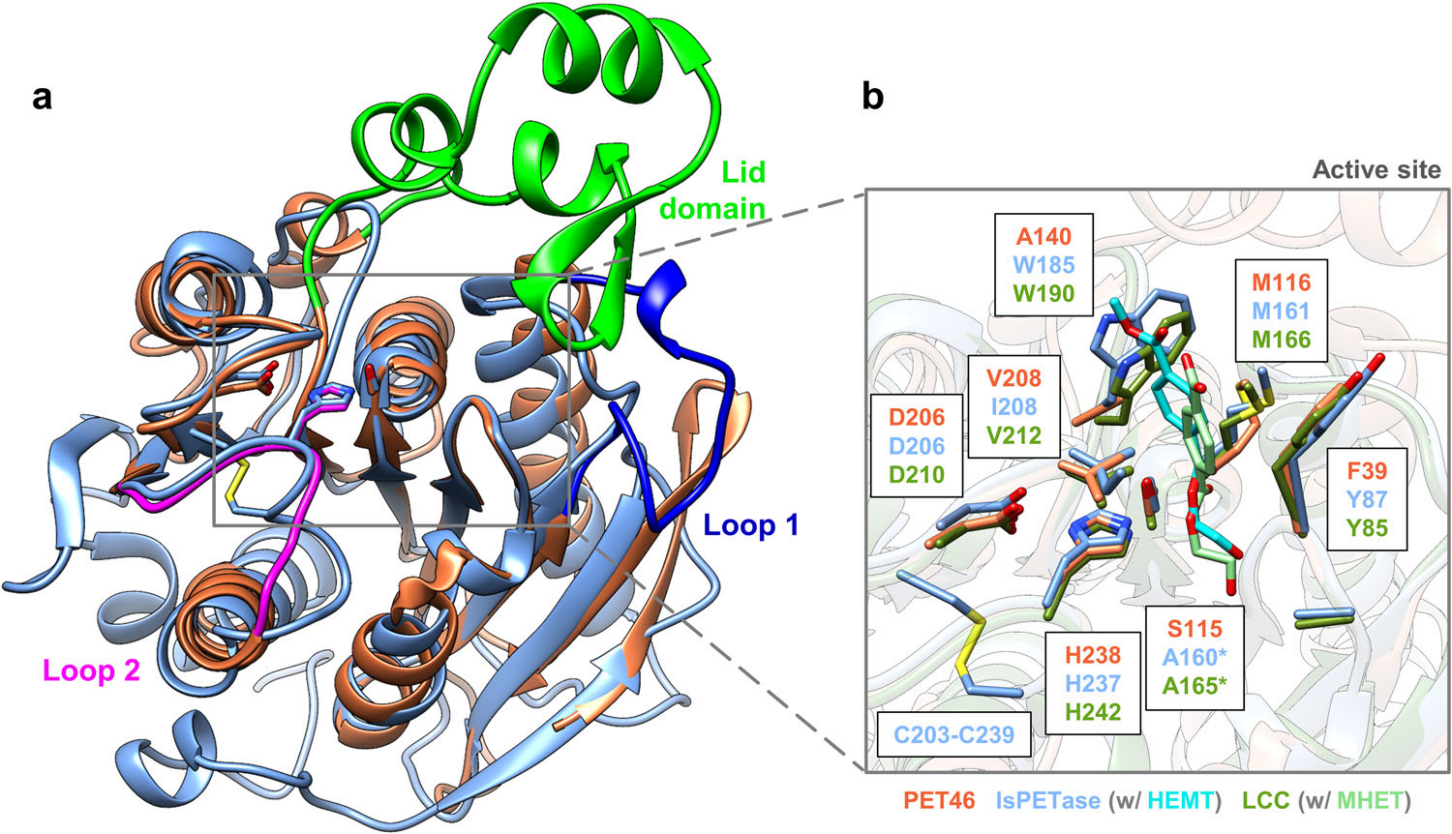
The crystal structure of PET46 resembles the crystal structure of the IsPETase and LCC - with unique features. All three proteins have the α/β-hydrolase fold and the same catalytic triad, but PET46 (coral orange; PDB 8B4U) contains a lid domain (bright green) that is not present in the IsPETase (sky blue; PDB 6EQE and 5XH3 in complex with HEMT - cyan) nor LCC (olive green, PDB 4EB0 and 7VVE in complex with MHET). Other structural differences are present in Loop 1 (deep blue) and Loop 2 (magenta), containing the active site’s His **a**. The bacterial and the archaeal enzymes present the typical residues of Ser-hydrolases at the catalytically active positions (Ser, His and Asp), but PET46 lacks a Trp associated with PET binding and formation of the aromatic clamp in the IsPETase and LCC. IsPETase is the only enzyme containing a disulfide bridge close to the active site **b**. Due to high similarity between IsPETase and LCC, and for clarity, only PET46 and IsPETase are shown in “a”. *5XH3 and 7VVE are inactive mutants.

One monomer is present in the asymmetric unit (ASU), which shares the common fold of the α/β-hydrolase superfamily, with the core of the enzyme being composed of eight β-strands connected by seven α-helixes (Supplementary Fig. 3). In addition, a lid domain composed of three α-helixes and two anti-parallel β-strands is present (Leu141-Val186). The active site is composed of the catalytic triad Asp206, His238, and Ser115, as inferred from structure homology to other α/β-hydrolases (Fig. 2). A phosphate ion and two ethylene glycol molecules were present near the active site (Supplementary Fig. 3), likely coming from the protein buffer, crystallization solution, and cryoprotectant.

Despite a low sequence identity of only 20% and 21%, respectively, the structure of PET46 overlays the well-studied IsPETase from *Ideonella sakaiensis* (PDB 6EQE) and LCC (PDB 4EB0) with 1.8 Å Cα-RMSD (Fig. 2a and Table 2). The largest difference is the medium-sized lid comprising 45 aa in PET46 (Leu141-Val186, Fig. 2a). Further structural differences around the active site are found in the enlarged loop between β4 and α3 (Loop 1; Asp68-Glu78; deep blue in Fig. 2a), which folds back to the outside, and the shorter loop between β10 and α10 containing the catalytic His (Loop 2; Arg234-Arg242; magenta in Fig. 2a). Loop 2 in IsPETase also contains one Cys that forms a disulfide bridge, which LCC and PET46 lack. Three out of four residues needed to form the oxyanion hole and the aromatic clamp are conserved or have equivalent properties as in IsPETase, LCC, and others PETases^13^. Nevertheless, the lack of an equivalent to Trp185 in IsPETase or Trp190 in LCC suggests that PET46 adopts a distinct substrate-binding pattern, probably involving the lid domain in the formation of the aromatic clamp (Fig. 2b). To answer this question, we constructed the chimera PET46ΔA140-P187::IsW185-T189 (from now on PET46Δlid), where we substituted Ala140-Pro187 with the homologous Trp185-Thr189 minimal loop of the IsPETase. By this, we included the Trp185 involved in the formation of the aromatic clamp, which is missing in PET46 (Fig. 2b). The produced mutant was verified by sequencing and SDS-PAGE, leading to a reduction in size of 5 kDa (Supplementary Fig. 2).

**Table 2:** PET46 has structural similarities to feruloyl esterases and bacterial PETases.

Protein Name	Protein Type	Host	Phylum	PDB	Z Score	RMSD [Å]	% Id	Source
PET46	PETase/FAE	Cand. Bathyarchaeota	Bathyarchaeota	8B4U	49.4	0.0	100	This work
GthFAE	FAE	*G. thermoglucosidasius*	Bacillota	7WWH	36.3	1.5	32	^95^
Est1E	FAE	*B. proteoclasticus*	Bacillota	2WTM	33.5	1.8	27	^42^
LJ0536	FAE	*L. johnsonii*	Bacillota	3PF8	32.8	1.9	28	^43^
202	PETase	*Chloroflexus sp*.	Chloroflexota	7QJM	32.3	2.2	27	^60^
306*	N/A*	*Cand. K. tengchongensis*	FCB	7QJN	30.6	1.9	25	^60^
703	PETase	*T. fusca*	Actinomycetota	7QJR	25.3	1.7	22	^60^
PET2	PETase	N/A**	Pseudomonadota**	7ECB	23.8	1.9	23	^25^
611	PETase	*S. flava*	Actinomycetota	7QJP	23.6	1.8	25	^60^
Thc_Cut2	PETase	*T. cellulosilytica*	Actinomycetota	5LUJ	23.5	1.7	22	^96^
606	PETase	*M. thermotolerans*	Actinomycetota	7QJO	23.4	1.8	20	^60^
TfH	PETase	*T. fusca*	Actinomycetota	5ZOA	23.3	1.6	22	PDB
TfCut_2	PETase	*T. fusca*	Actinomycetota	4CG1	23.2	1.7	22	^97^
705	PETase	*T. fusca*	Actinomycetota	7QJS	23.2	1.7	22	^60^
702	PETase	*T. fusca*	Actinomycetota	7QJQ	23.2	1.7	22	^60^
Cut190	PETase	*S. viridis*	Actinomycetota	4WFK	23.1	1.9	20	^98^
PbauzCut	PETase	*P. bauzanensis*	Pseudomonadota	8AIT	23.1	1.8	25	^99^
Est119	PETase	*T. alba*	Actinomycetota	6AID	23.0	1.7	22	^100^
IsPETase	PETase	*I. sakaiensis*	Pseudomonadota	6EQE	23.0	1.8	23	^101^
PHL-7	PETase	N/A**	Actinomycetota**	7NEI	22.9	1.9	21	^28^
711	PETase	*T. cellulosilytica*	Actinomycetota	7QJT	22.9	1.7	22	^60^
PLE629	PETase	Marinobacter sp.	Pseudomonadota	7VPA	22.9	1.8	23	^102^
Thc_Cut1	PETase	*T. cellulosilytica*	Actinomycetota	5LUI	22.9	1.7	22	^96^
BhrPETase	PETase	Bacterium HR29	Actinomycetota**	7EOA	22.7	1.8	21	^103^
RgCut-II	PETase	*R. gummiphilus*	Pseudomonadota	8AIR	22.7	1.8	23	^99^
LCC	PETase	N/A**	Actinomycetota**	4EB0	22.6	1.8	23	^22^
RgPETase	PETase	*R. gumimpihilus*	Pseudomonadota	7DZT	22.4	1.8	23	^104^
PET6	PETase	*V. gazogenes*	Pseudomonadota	7Z6B	22.1	1.9	22	^105^
PET30	PETase	*K. jeonii*	Bacteroidota	7PZJ	21.9	1.9	23	^29^
PE-H	PETase	*P. aestusnigri*	Pseudomonadota	6SBN	21.5	2.1	25	^24^
PsCut	PETase	*P. saudimassiliensis*	Pseudomonadota	8AIS	20.8	2.2	25	^99^
PLE628	PETase	Marinobacter sp.	Pseudomonadota	7VMD	20.2	2.1	26	^102^
PmC	PETase	*P. mendocina*	Pseudomonadota	2FX5	19.9	2.2	19	^106^
CalB	Lipase	*C. albicans*	Ascomycota	1TCA	16.6	2.9	12	^31^
IsMHETase	MHETase	*I. sakaiensis*	Pseudomonadota	6QZ4	14.6	3.2	13	^41^
HiC	PETase	*T. insolens*	Ascomycota	4OYL	12.9	3.1	12	^31^
FsC	PETase	*F. solani*	Ascomycota	1AGY	12.7	3.2	11	^31^

**Fig. 3:**
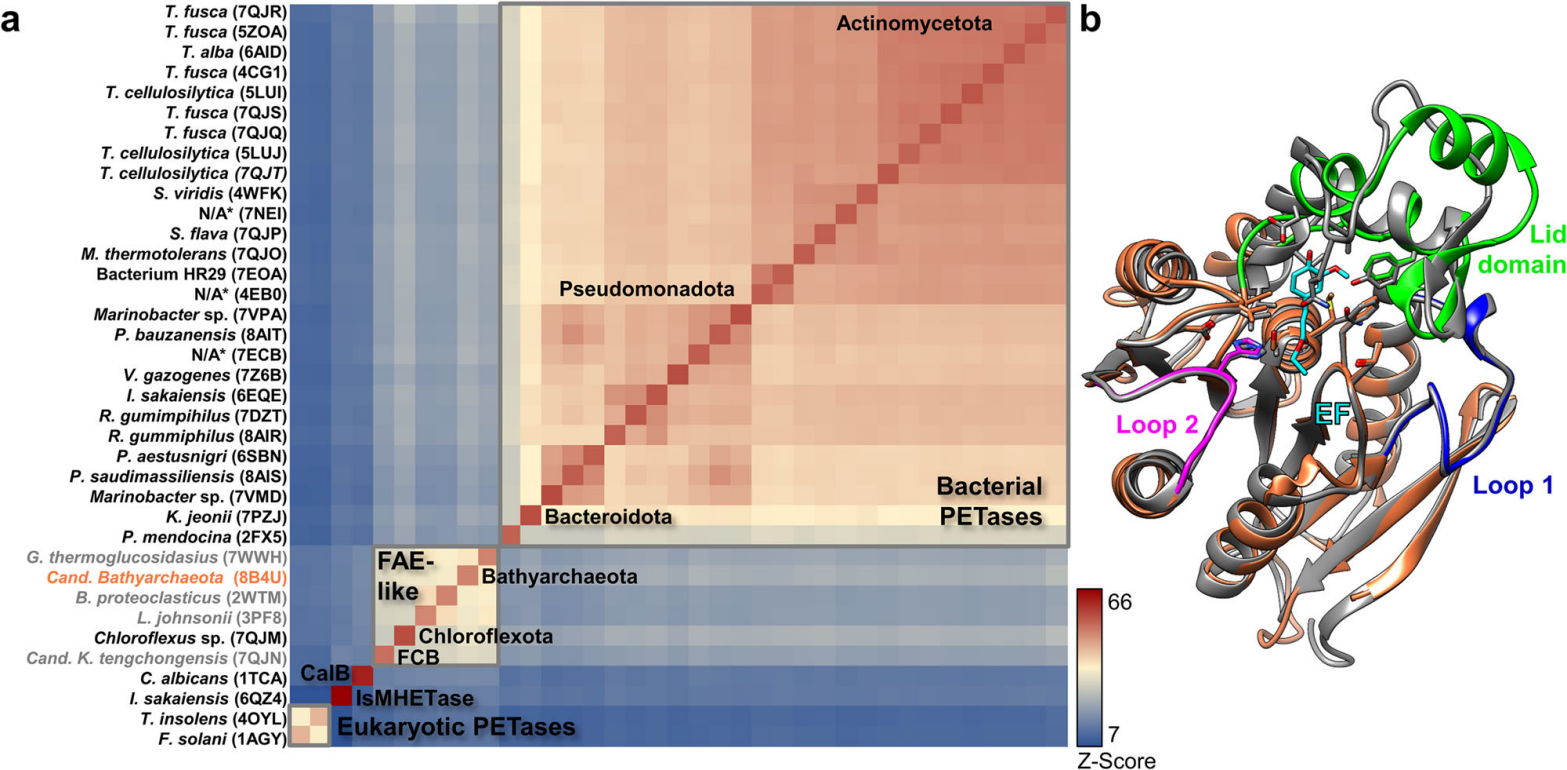
The protein structure of archaeal PETase PET46 and ferulic acid esterases (FAEs) is closely related to bacterial PETases. A heatmap represents structure similarity (Z-Score^87^) and reveals structural clusters. The FAE cluster, to which PET46 (coral orange) belongs, shows the highest similarity to the cluster of bacterial PETases. PET 46 is the FAE with the highest structural similarity to the bacterial PETases. Enzymes for which no activity on PET has been proven (*e.g*., FAEs) are shown in gray **a**. PET46 shares most of its structure with FAEs **b**. The structure of the archaeal PETase (coral orange) is overlaid to the crystal structure of the cinnamoyl esterase LJ0536 S106A mutant from Lactobacillus johnsonii (dark gray, PDB 3QM1) in complex with ethylferulate (EF, cyan). Loop 1 (deep blue) and Loop 2 (magenta) are highly conserved, but there are some variations in the lid domain (bright green). A Tyr in the loop of LJ0536 involved in substrate binding has a homologous Phe in PET46 (bright green). For structural alignments with other two FAEs and the tannase IsMHETase, see Supplementary Fig. 4. *No obvious phylogenetic affiliation. Data supporting panel “a” can be found in Supplementary Data 2.

Crystal structures included in the analysis in Fig. 3 are sorted according to their Z-Score^87^ compared to PET46. *FAE* Ferulic Acid Esterase/Feruloyl Esterase. *No PETase activity detected. **Phylogeny could not be inferred.

We further compared the structure of PET46 to all published bacterial and eukaryotic PETase crystal structures (Table 2). Additionally, we performed searches against all crystal structures in the PDB. From this database, the best hits obtained were the feruloyl esterases GthFAE from *Geobacillus thermoglucosidasius* (PDB 7WWH) and Est1E from the rumen bacterium *Butyrivibrio proteoclasticus* (PDB 2WTM) together with the cinnamoyl esterase LJ0536 from *Lactobacillus johnsonii* (PDB 3PF8; Fig. 3a and Table 2). All hits derive from Firmicutes. Feruloyl esterases are also known as ferulic acid esterases (FAEs). They are involved in plant biomass degradation and cleave *e.g*., cinnamic, *p*-coumaric or ferulic acid, thus decoupling plant cell wall polysaccharides and lignin^39^. Using ethyl cinnamate (EC) as a model substrate, we could detect enzyme-mediated H^+^ release derived from ester hydrolysis (Supplementary Fig. 4). These aromatic acids esterified to long polymers may be analogous to MHET units at the end of a PET chain (Fig. 3b and Supplementary Fig. 4). FAEs are believed to be secreted enzymes, even if no apparent N-terminal signal peptide is present^40^. In the case of PET46, no obvious secretion signal is detected. Since FAEs form a protein family with tannases, to which the MHETase from *I. sakaiensis* belongs^41^, we also included its structure (PDB 6QZ4, Table 2) in our structural analysis.

PET46 and all three FAEs shared the highest structural similarity. Even Loop 1 and Loop 2 are highly conserved, but some variations are observed at the lid domain (Fig. 3b, Table 2, and Supplementary Fig. 5). PET46 and GthFAE share the G-L-S-M-G motif, similar to the bacterial PETase´s G-W/H-S-M-G. The other two FAEs have G-H-S-Q-G, similar to eukaryotic PETase´s G-Y-S-Q-G. We analyzed the crystal structures of Est1E and LJ0536 co-crystallized with ferulic acid (FA; PDB 2WTN) or ethyl-ferulate (EF; PDB 3QM1) and identified up to five positions in the lid that could be involved in substrate binding, including four aromatic Tyr, Phe, or Trp residues^42,43^ (1–4 in Supplementary Fig. 3a). The only conserved aromatic position in the lid of all FAEs is PET46’s Phe178 (3 in Supplementary Fig. 3a); Phe in GtfFAE and Tyr in the other two (Fig. 3b, Supplementary Fig. 5). Overall, PET46 and FAEs build a cluster that is most similar to the cluster formed by bacterial PETases (Fig. 3a). The archaeal PETase is structurally most similar to the metagenomic bacterial PETase LipIAF5-2 (PET2^25^, Table 2). We then proceeded to characterize PET46 biochemically to confirm PETase activity.

### PET46 is a promiscuous feruloyl esterase that hydrolyzes MHET, BHET, 3PET, and PET polymers

We tested PET46 for its activities on bis-(2-hydroxyethyl) terephthalate (BHET) and mono-(2-hydroxyethyl) terephthalate (MHET). Subsequently, we assayed activities on PET trimer (3PET) and polymers, both powder and foil. This clearly showed that PET46 can break down plastic PET and the not fully hydrolyzed degradation products.

**Fig. 4:**
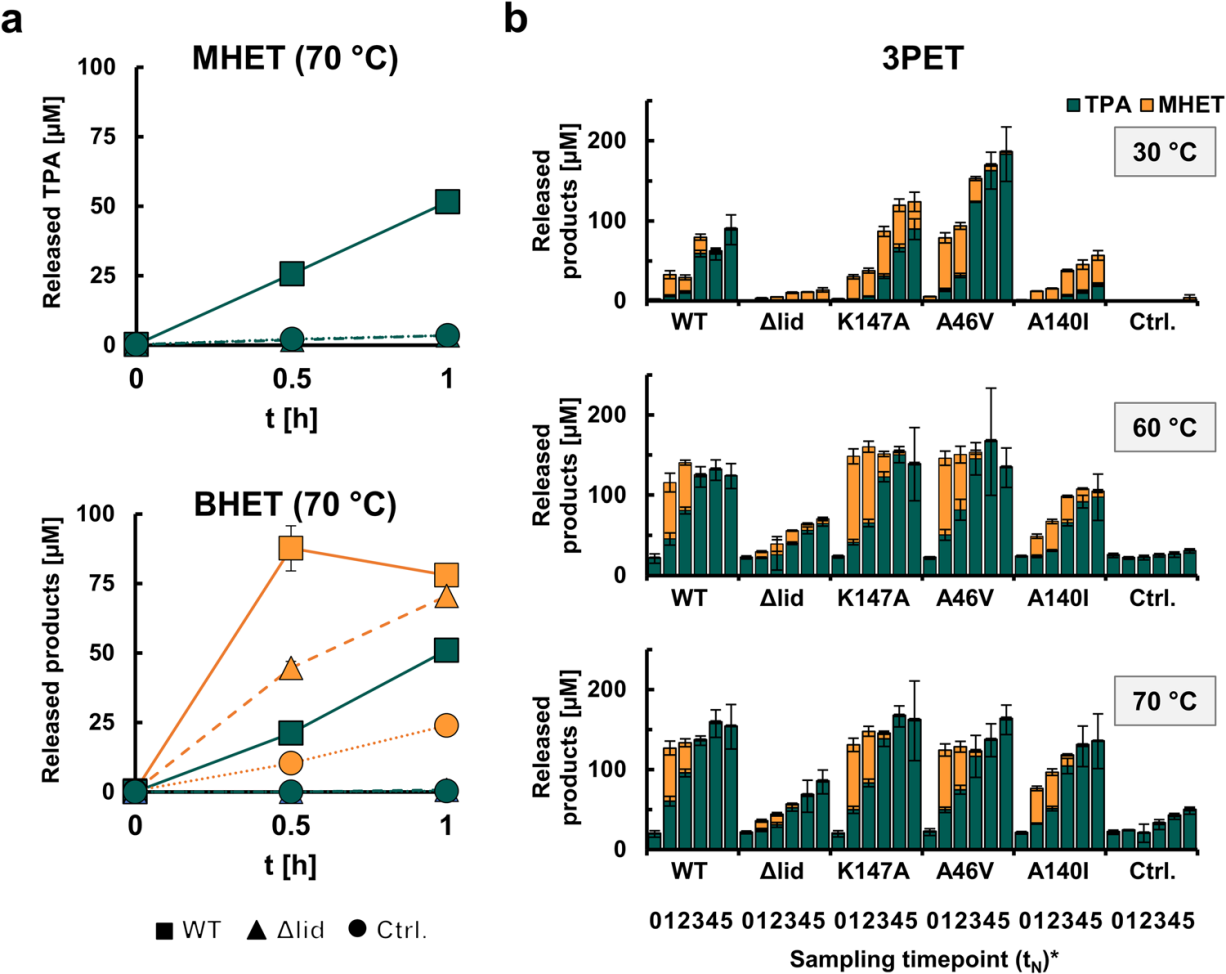
The lid domain of PET46 is involved in effective degradation of MHET, BHET and 3PET. PET46 WT can degrade both BHET and MHET to TPA and EG at 70 °C, but the lidless variant PET46Δlid can only convert BHET to MHET (orange, **a**). PET46 and the produced variants degrade 3PET at 30, 60, and 70 °C **b**. All experiments contain 3 µM enzyme and 150 µM TPA equivalents in 200 µL 0.1 M potassium phosphate buffer pH 8. Error bars indicate the standard deviation (*n* = 3). *t_0_ = 0 h; t_1_ = 3 h; t_2_ = 6 h; t_3_ = 24 h; t_4_ = 48 h; t_5_ = 72 h. Supporting data can be found in Supplementary Data 2.

PET46 WT can degrade both BHET and MHET. In less than 30 min, all BHET (150 µM in 200 µL) was converted to MHET and terephthalic acid (TPA) in a 4:1 ratio. After 1 h of incubation, 50.99 µM TPA were measured. Incubation with the same amount of MHET for 1 h resulted in 51.5 µM TPA released (Fig. 4a), implying that MHET degradation occurs at the maximum rate independent from the starting substrate. PET46Δlid could release up to 70.53 mM MHET from BHET, but we did not detect MHET degradation within 1 h incubation (Fig. 4a). Thus, the lid may be involved in efficient substrate binding and catalysis.

To better understand the BHET-degrading ability of PET46, its crystal structure was applied in docking experiments with this substrate. Two main clusters of docked poses were obtained, covering 83% and 12% of all solutions, and two smaller clusters containing 4% and 1% (Supplementary Fig. 6). For both main clusters, the smallest distance between the substrate’s carbonyl carbon and the hydroxyl oxygen from the catalytic serine is below 3.1 Å, indicating a plausible orientation of the substrate’s ester group towards the catalytic nucleophile (Supplementary Fig. 6). We found that for both main clusters, the backbone amides of Phe39 and Met116 form an oxyanion hole that potentially stabilizes the transition state during ester hydrolysis (Supplementary Fig. 7). Furthermore, we compared the potential binding poses of BHET within PET46 to the binding pose of ethyl-ferulate (EF) within the crystal structure of the cinnamoyl esterase LJ0536 S106A mutant (PDB 3QM1). The docking pose identified in the second cluster is very similar to the position of the EF, especially with respect to the positioning of the carbonyl moiety (Supplementary Fig. 8). Likewise, this cluster adopts an analogous position as in the crystal structures of IsPETase and LCC^ICCG^ co-crystallized with HEMT^44^ and MHET^45^ (5XH3 and 7VVE; Fig. 2b). Thus, we propose that as the most probable.

We asked if any other position was involved in substrate accommodation or product release. Based on the docking results, we identified two amino acids, Ala46 and Ala140, near both predominant docking poses that might be relevant for the substrate accessibility and binding (Supplementary Fig. 6). Introducing the larger substitutions A46V and A140I should thus impact the catalytic activity. We further identified Lys147, which possibly interacts with docked poses from the second-largest cluster. Variant K147A abolishes this interaction and widens the binding groove (Supplementary Fig. 6). Note that the lid of PET46 has potentially adopted a non-optimal conformation/orientation for substrate binding. Therefore, docking into the rigid crystal structure might underestimate the potential importance of the flexible lid for ligand binding.

We then proceeded to incubate PET46 WT and all the constructed variants (including PET46Δlid) on 3PET at 60 and 70 °C (optimal temperature range, see next chapter) and 30 °C as a mesophilic control. At the two highest temperatures, we observed a similar activity pattern, where PET46 WT, K147A, and A46V degraded all the 3PET to MHET and TPA within the first 3 h (Fig. 4b). PET46 A140I performed slightly worse, while PET46Δlid could only convert half of the 3PET after 72 h incubation (Fig. 4b). Interestingly, A46V produced 3.2 times more products at 30 °C than the WT enzyme during the first 24 h. In all experiments, we were not able to detect any BHET. Together with the previously obtained BHET and MHET degradation activities, and the fact that PET46 is a FAE, we assume hydrolysis happens at the polymer chain end (exo-activity), where 3PET is quickly cleaved to MHET units, which are subsequently converted to TPA and ethylene glycol (EG). This method of action has been proposed and experimentally shown recently for another 3PET-degrading esterase^46^, although their structures differ substantially (Supplementary Fig. 5d).

**Fig. 5:**
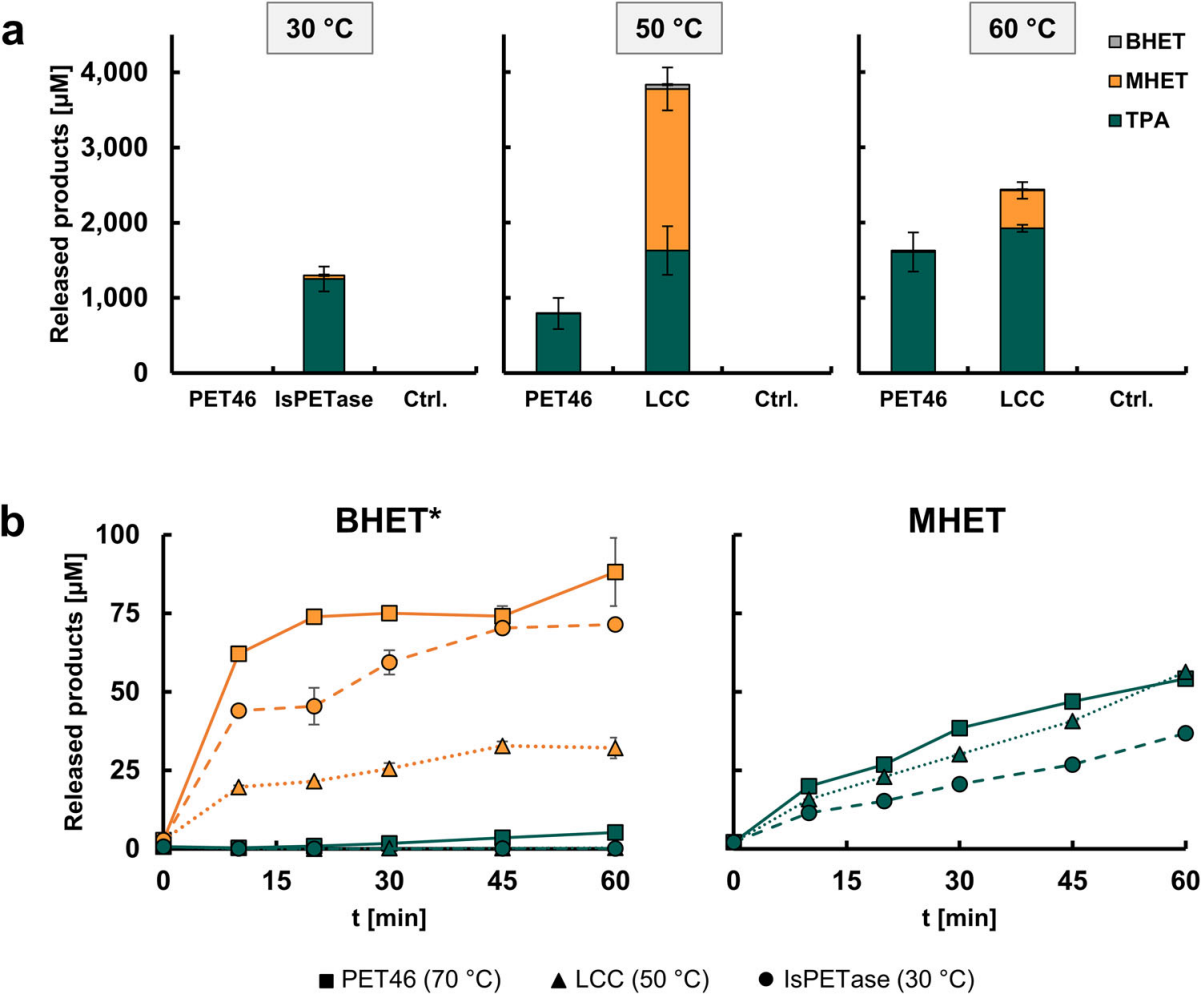
PET46 degrades PET polymer. PET46’s PET-degrading ability was assayed at 60 °C and compared to the IsPETase and the LCC at their respective temperature optima (30 °C and 50 °C; **a**). 3 µM PET46 (roughly 0.1 mg mL^−1^) release up to 1.6 mM TPA out of 48 mM crystalline PET powder after 72 h at 60 °C. PET46 is more effective than IsPETase and LCC in degrading the intermediates BHET and MHET (150 mM) at their respective optimal temperatures **b**. The color palette is shared between “a” and “b”. Product release linked to autohydrolysis at the different temperatures was subtracted at each timepoint. Enzyme concentrations were kept equal for all assayed enzymes within each experiment. Data represent mean results (*n* = 3). Error bars indicate standard deviation. *Due to faster degradation of BHET by all enzymes, enzyme concentration used is 50× less than for PET and MHET degradation. Supporting data can be found in Supplementary Data 2.

Finally, we assayed PET46 WT on semi-crystalline PET powder and amorphous film. We could not detect any activity on PET foil. Surprisingly, incubation of 3 µM enzyme (1 mg mL^−1^) with semi-crystalline PET powder released up to 1,624.14 µM aromatic products (TPA, MHET and BHET) in 200 µL after three days at 60 °C, more than 99.1% being TPA (Fig. 5a and Table 3). This corresponds to 3.38% total conversion. We compared the archaeal enzyme to the best-characterized IsPETase and LCC WT enzymes at their respective temperature optima (30 and 50 °C; Fig. 5a and Table 3). At 30 °C, PET46 released virtually no aromatic products, while incubation of PET powder with IsPETase resulted in 1,299.97 µM products (96.2% TPA and 2.71% conversion). Since we did observe activity on 3PET at said temperature (Fig. 4b), we assume that PET46 needs high temperatures to attack true PET polymer. At 50 °C, PET46 released 791.40 µM products (99.9% TPA and 1.65% conversion), where incubation with LCC lead to the release of 3,832.67 µM (7.98% conversion and five times more than PET46). Interestingly, TPA amounts released by LCC account for only 42.46% of the total (TPA:MHET:BHET ≈ 1:1.32:0.04). At 60 °C, the PETase activity of LCC decreases by 36.3%, releasing 2,440.68 µM aromatic compounds (78.8% TPA and 5.08% conversion). This makes LCC WT only 33.4% more active than PET46 at 60 °C. Further experiments revealed that most product release happens during the first 24 h of incubation and supplementation with fresh enzyme (fed-batch) does not promote additional hydrolysis (Supplementary Fig. 9). This suggests again that the enzyme is most probably just cleaving terminal TPA moieties. Initial experiments showed that PET46Δlid did not hydrolyze PET polymer. Nevertheless, fed-batch incubations resulted in a slight degradation after 2 days (Supplementary Fig. 9).

**Table 3:** PET46 degrades semi-crystalline PET powder at high temperatures.

Enzyme	T [°C]	TPA [µM]	MHET [µM]	BHET [µM]	Σ [µM]	Conv. [%]
PET46		1.91 ± 0.77	0 ± 0	0 ± 0	1.91 ± 0.77	0.00
IsPETase (*)	30	1250.56 ± 166.54	49.41 ± 11.23	0 ± 0	1,299.97 ± 166.91	2.71
Ctrl.		3.75 ± 9.44	0.35 ± 0.3	0.13 ± 0.04	4.23 ± 9.45	0.01
PET46		790.41 ± 206.22	0.85 ± 0.82	0.14 ± 0.06	791.4 ± 206.22	1.65
LCC (*)	50	1627.57 ± 322.23	2147.9 ± 286.1	57.21 ± 8.3	3,832.67 ± 430.99	7.98
Ctrl.		6.27 ± 7.89	0.68 ± 0.14	0.14 ± 0.03	7.09 ± 7.89	0.01
PET46 (*)		1609.25 ± 260.17	14.44 ± 2.07	0.45 ± 0.13	1,624.14 ± 260.18	3.38
LCC	60	1922.12 ± 47.76	505.29 ± 110.34	13.26 ± 3.46	2,440.68 ± 120.28	5.08
Ctrl.		7.37 ± 1.02	0.86 ± 0.18	0.2 ± 0.09	8.44 ± 1.04	0.02

Product release measurements underlying Fig. 5a show that the WT enzymes PET46, IsPETase, and LCC release comparable amounts of aromatic compounds after three days at their optimal temperatures (indicated with an asterisk). Percentage of conversion assumes an initial concentration of 48 mM TPA equivalents.

As for the incubation with 3PET, we did not detect any BHET or only trace amounts, and MHET was also a minor product (Table 3). We asked how the BHETase and MHETase activities compared to the reference enzymes, for which we incubated them with both substrates at their respective optimal temperature (Fig. 5b). After only 10 minutes at 70 °C, 0.06 µM PET46 degraded 62.06 µM BHET to MHET and EG (starting concentration 150 µM in 200 µL). Under the same conditions but at 30 and 50 °C, respectively, IsPETase and LCC degraded only 44.03 and 19.66 µM (Fig. 5b). This makes PET46 40.9% and 215.7% more active on this substrate. On MHET, 3 µM PET46 releases 38.44 µM TPA after 30 min at 70 °C. LCC and IsPETase release 30.11 and 20.63 µM, respectively (Fig. 5b). PET46 is therefore up to 27.7 and 86.3% more active on MHET than the reference enzymes. U mg^−1^ values are presented in Table 4. We speculate that the lid domain, not present in the other PETases, is involved in the better accommodation of these substrates in the active site (Supplementary Fig. 6).

**Table 4:** Enzyme activity of PET46, LCC, and IsPETase on BHET and MHET.

Enzyme	Activity on BHET [U mg^−1^]	Activity on MHET [U mg^−1^]	Ratio BHET:MHET
PET46	3.103	0.020	155.85
LCC	2.202	0.016	139.87
IsPETase	0.983	0.011	85.78

One U is defined as µmol min^−1^. For IsPETase, mg refers to the fraction of the enzyme that does not contain the maltose-binding protein (MBP).

### PET46 is adapted to the geochemical conditions at the Guaymas Basin

We characterized PET46 in more detail and with respect to its temperature and substrate profile. Therefore, a substrate spectrum was recorded with *p*NP-esters, which had an acyl chain length of 4 to 18 C-atoms. The highest activities of PET46 were observed with *p*NP-decanoate (C10). PET46 was only poorly active on short (C4-C6) and long (C12-16) acyl chain lengths (Supplementary Fig. 10).

**Fig. 6:**
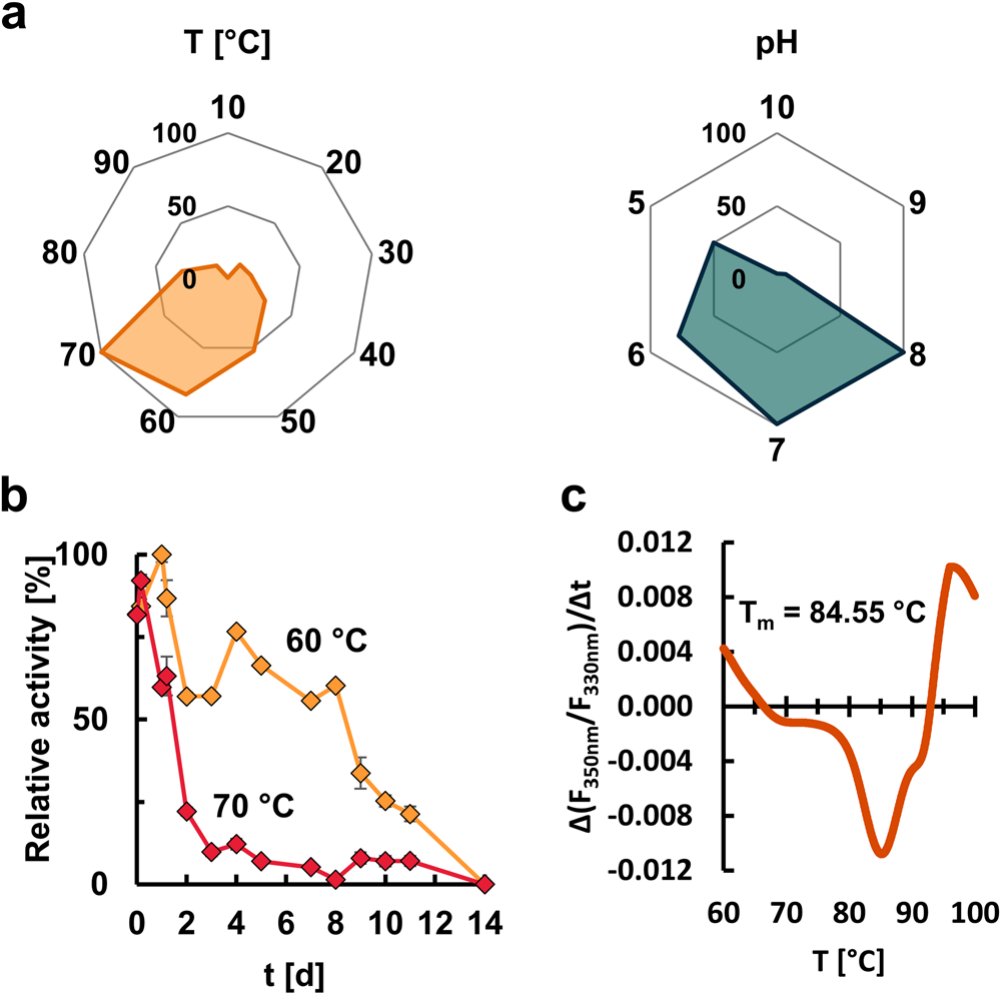
PET46 is a thermostable hydrolase adapted to the conditions of the Guaymas Basin. The enzyme’s optimal temperature and pH were determined by incubation with *p*NP-ester substrates (decanoate, C10, **a**). The enzyme conserved most of its activity after 8-day incubation at 60 °C, but lost almost 80% after two days at 70 °C **b**. NanoDSF experiments show that PET46 has a melting temperature (T_m_) of 84.55 °C **c**. Error bars indicate the standard deviation (*n* = 3). Standard deviation in “a” was below 6% for all conditions assayed. Supporting data can be found in Supplementary Data 2.

Using 1 mM *p*NP-decanoate as substrate, the recombinant enzyme PET46 revealed a broad temperature spectrum. The highest activity was observed at 70 °C, while at 90 °C only 10% residual activity was detectable. The enzyme remained active at a temperature below 40 °C, but only had low activities (Fig. 6a). To further assess thermostability, the recombinant PET46 was incubated at 60 °C and 70 °C for two weeks. At 60 °C, the enzyme kept more than 60% of its activity for up to 8 days. At 70 °C, 80% of the activity was lost after 2 days, with only 10% remaining after 3 days (Fig. 6b). NanoDSF experiments measured a T_m_ of 84.55 °C (Fig. 6c). The original metagenomic sample was collected at a temperature of 48 °C^34^, at which PET46 shows 52% relative activity under laboratory conditions.

PET46 revealed activity at a broad pH range of 5–8. It had its optimum at pH 7–8 when tested on 1 mM *p*NP-C10 at 70 °C. However, it also retained high activities (50%) at pH 5 (Fig. 6a). The pH at the Guaymas Basin is recorded to be approximately 5.9^47^, at which PET46 would exhibit up to 77% of its activity under laboratory conditions.

To further characterize the effects of various metal ions, PET46 was incubated for 1 h with 1 or 10 mM Ca^2+^, Co^2+^, Cu^2+^, Fe^3+^, Mg^2+^, Mn^2+^, Ni^2+^ or Zn^2+^. The activity was assayed under optimal conditions and compared to a metal-free control. The activity of PET46 significantly increased in the presence of most of these ions. In contrast, Cu^2+^ reduced the activity by 50%. Especially the addition of Zn^2+^ resulted in an almost two-fold activity increase (Supplementary Fig. 10). Some of these ions are present at significant concentrations in the Guaymas Basin^48^. Thus, metal binding to the protein seems plausible.

Further, we tested the sensitivity of PET46 towards detergents and the reducing agent DTT. A concentration of 1 and 5% of the detergents Triton X-100 and DTT strongly affected the enzyme activities (Supplementary Fig. 10). Interestingly, 1% DTT stimulated esterase activity by a factor of two.

Finally, we assessed the solvent tolerance of PET46. In general, the enzyme was remarkably stable with acetone, DMF, isopropanol, and DMSO. Notably, 10% acetone and 5% DMSO and DMF increased the enzyme’s activities by a factor of 2 (Supplementary Fig. 10). This is a noteworthy solvent tolerance.

Overall, PET46 is a well-adapted and very stable enzyme in its natural environment. Bathyarchaeota are ubiquitous and the predominant archaea in deep-sea environments like the Guaymas Basin^36,49^, and they have been shown to grow on lignin as energy source^50^, for which enzymes like PET46 need to be secreted. Together with our results on PET poly-, oligo- and monomer hydrolysis, we conclude that enzymes associated with lignin degradation, and especially FAEs from Bathyarchaeota and other prokaryotes, may have a global impact in promiscuity-driven degradation of PET litter in the deep-ocean, even if degradation happens only at low rates.

## Discussion

The degradation of polyethylene terephthalate (PET) through enzymatic hydrolysis has gained considerable attention as a promising approach to address the global plastic waste crisis. PET-degrading enzymes, including cutinases (EC 3.1.1.74), lipases (EC 3.1.1.3), and carboxylesterases (EC 3.1.1.1), which are known to be very promiscuous^51,52^, target the ester bond within the amorphous or low-crystalline regions of the polymer, leading to the production of key intermediates BHET, MHET, as well as TPA and EG^16^.

Mesophilic enzymes such as IsPETase have been shown to hydrolyze PET at relatively low temperatures^26^. This finding challenged the common misconception that PET degradation is solely efficient at around the glass transition temperature (Tg) of PET, often believed to be 70 °C^33^. A recent study demonstrated that, when exposed to water, the T_g_ of the thin surface layer decreases to approximately 40 °C^53^. Nevertheless, thermostability is a notable characteristic of many highly active PETases, with optimal temperatures ranging from 55 to 65 °C. These enzymes are best-suited for industrial applications, where elevated temperatures can enhance reaction rates and improve overall process efficiency, but this phenomenon is nevertheless only explained by the Arrhenius equation^53^. In light of these findings, much work has been done to improve current PETases regarding their heat-resistance^33,54–59^. But there is also a growing need to expand our knowledge of heat-stable enzymes and explore new protein scaffolds that can further optimize PET degradation^60^. Furthermore, mining the biodiversity can also help understand the global distribution and roles of these promiscuous enzymes in nature.

Within this setting, we provide compelling evidence that *Candidatus* Bathyarchaeota archaeon B1_G2 hosts the promiscuous esterase PET46 that can act on semi-crystalline PET polymer. Besides, PET46 hydrolyzes BHET and MHET with significant rates, confirming that it can handle both the polymer and the intermediates (Figs. 4 and 5). Based on structural analysis and activity assays (Fig. 3 and Supplementary Fig. 4), PET46 is a feruloyl esterase. Feruloyl/ferulic acid esterases (FAE; EC 3.1.1.73) release ferulic acid and other hydroxycinnamic acids from plant-based hemicellulose and lignin, which have a large biotechnological application^61^. They are widespread in nature and have been found in bacteria, plants, and fungi. Their 3D structure usually reveals a canonical eight-strand α/β-fold of lipases and esterases (Supplementary Fig. 3). In addition, a lid domain is observed, which, analogous to lipases, confines the active site with a loop that confers plasticity to the substrate-binding site^62^.

Analogous to PETases, FAEs remove aromatic acids from the end of polymers (Supplementary Fig. 4). A recent study described a metagenomic FAE with phthalate-degrading activity, but no PET degradation was assayed^63^. PETases are assumed to not have a lid domain. However, some enzymes acting on the intermediate MHET are annotated as tannases, which form a protein family with FAEs, and they bear a lid domain of varying length. They can hydrolyze MHET into TPA and EG. One of the best-studied examples is the MHETase derived from the gram-negative bacterium *I. sakaiensis* (IsMHETase). This enzyme acts efficiently on MHET, but recently an exo-function on PET pentamers was described^64^. It also hydrolyzed BHET in a concentration-dependent manner, and its three-dimensional structure shows a much larger lid domain involving more than 200 aa (Supplementary Fig. 5). A new study by Erickson et al.^60^ searched for new thermotolerant PET hydrolase scaffolds following a similar searching strategy as in our study. They reported the metagenome-derived enzymes 204 and 211, but did not investigate their taxonomic affiliation nor their conserved domains in detail. PETase 204 is 97.93% identical to WP_187147021.1 and WP_148684612.1 from *Thermophilum adornatum*, while 211 is 99.62% identical to MBS7639053.1 from a Cand. Bathyarchaeota archaeon, both archaea. PET46 shares 34.92% and 65.78% sequence identity, respectively (Fig. 1), but their structure is very similar (Supplementary Fig. 11). In their study, the PET46-like 211 released up to 85.23 mg L^−1^ aromatic products from crystalline PET powder at 60 °C and pH6 (optimal conditions). This equals to 0.52 mM assuming all of it is TPA, which is less but in the range of the amount of product that we measured with PET46 (1.6 mM; Fig. 4). As for PET46, no activity was detected on amorphous PET foil.

PET46 is encoded in a marine Bathyarcheota MAG. Microorganisms affiliated with the Bathyarchaeota are globally occurring and widespread in marine and terrestrial anoxic sediments^65^. They can use a wide range of polymers as carbon and energy sources, and they are well known to be very versatile with respect to metabolic capabilities. They are further known to be abundant in some marine sediments. Because of their huge metabolic potential, it is further assumed that they may play a significant role in global carbon biogeochemical cycling^65–68^. Interestingly, Bathyarcheota have been associated with the degradation of the biopolymer lignocellulose previously^50^. Therefore, the observation here that not only their genomes code for FAEs, but also the demonstration that they are functionally active underscores this observation.

Within this setting, PET46 being catalytically active on PET powder is in line with the known wide metabolic diversity of the Bathycharchaeota^69^. When we benchmarked our enzyme with the well-characterized enzymes IsPETase and LCC (WTs), our data imply that the overall PETase activities observed for PET46 are comparable (Fig. 5). Furthermore, PET46 was faster in the degradation of intermediates BHET and MHET. We expect that evolution experiments would enhance the activity of PET46, a necessary step to make the enzyme a viable candidate for possible industrial application^46,70^.

While PET esterases are not highly conserved among each other, few structural traits and sequence homologies are common in most of the known enzymes (Fig. 3). Based on our data analyses and others^33^, it becomes evident that most PETases do not carry a lid domain, which was proved to be crucial for enzymatic activity of PET46 (Fig. 4 and Supplementary Fig. 9). All published active enzymes are secreted proteins that carry at least an N-terminal signal peptide and some even a PorC-like type 9 secretion system (T9SS) domain^29^. The region involved in substrate binding (formation of the aromatic clamp and oxyanion hole) contains in general the amino acids Tyr/Phe-Met-Trp/Tyr, and the catalytic triad is composed of Asp-His-Ser. Further, active enzymes carry 1-2 disulfide bonds and of these, one is close to the active site (Fig. 2b). The active site is well accessible for bulky substrates and is in a large cavity. For more detailed analyses of common PETase features, we refer to other studies^13,33^.

In summary, our biochemical results significantly extend the knowledge of PETase enzymes and their biodiversity. Our study further enables the development of an expanded phylogenetic framework for identifying the diversity of putative PET-degrading enzymes in marine microbial groups throughout the global ocean. Finally, the data presented here will help advance our knowledge on the ecological role of the Bathyarchaeota and the possible decomposition of marine PET litter.

## Methods

### Profile Hidden-Markov Model (HMM) searches identify putative archaeal PETases

An HMM constructed from all PET-degrading enzymes listed in the PAZy database^16^ was used to search against NCBI’s non-redundant protein database^71^ (ftp.ncbi.nlm.nih.gov/blast/db/FASTA/nr.gz) filtered for sequences of archaeal origin (tax ID: 2157), as described previously^13,25,35^. In short, a multiple sequence alignment (MSA) was performed with T-Coffee v.12.00 in Expresso mode^72^ and a profile HMM was constructed with hmmbuild and applied on a search with hmmsearch from the HMMER v.3.3 software package^73^.

### Primers, constructs, and bacterial strains used

The gene coding for PET46 was codon-optimized and synthesized in pET21a(+) (Novogene, Cambridge, UK) by Biomatik (Ontario, Canada) and transformed in *Escherichia coli* BL21(DE3) (Novagen/Merck, Darmstadt, Germany) for protein production. The primers used to generate all PET46 mutants by site directed mutagenesis were synthesized by Eurofins Genomics (Ebersberg, Germany), and are listed in Supplementary Table 1. Sequencing of all constructs was conducted by Microsynth Seqlab GmbH (Göttingen, Germany).

### Protein production and purification

PET46 WT, its mutant derivatives, and LCC were produced heterologously by growing *E. coli* BL21(DE3) cells carrying the respective pET21a(+) construct at 37 °C in Lysogeny Broth/Luria-Bertani (LB) medium containing 100 µg mL^−1^ ampicillin. When OD_600_ reached 0.7, 1 mM IPTG was added to induce expression of the genes and cultures were incubated overnight at 22 °C to facilitate protein production. The IsPETase from *I. sakaiensis* was produced in a similar manner, but the vector was the pMAL-p4X. Cells were centrifuged and lysis was carried out via French Press three times at 1,250 psi. The proteins were purified from the cleared lysate with Ni-NTA agarose (Macherey-Nagel, Düren, Germany) or maltose columns, following concentration and dialysis against 0.1 M potassium phosphate buffer pH 7.

### Crystallization, data collection, data reduction, structure determination, refinement, and final model analysis

PET46 was crystallized by sitting-drop vapor-diffusion at 12 °C with a concentration of 10 mg mL^−1^ in 100 mM potassium phosphate buffer pH 7. 1.5 µL of PET46 were mixed with 1.5 µL of reservoir solution consisting of 325 mM (NH_4_)H_2_PO_4_. Crystals formed after 3–4 weeks, were harvested, and cryo-protected with 35% ethylene glycol followed by flash-freezing in liquid nitrogen. Diffraction data were collected at −173 °C (100 K) at beamline ID23-1 (ESRF, Grenoble, France) using a 0.9793 Å wavelength. Data reduction was performed using XDS^74^ and Aimless^75^ from the CCP4 Suite^76^. The structure was solved via molecular replacement with Phaser^77^ using an AlphaFold^78^ model as search model. The initial model was refined alternating cycles of manual model building in COOT^79,80^ and automatic refinement using Phenix^81^ v.1.19.2_4158. Data collection and refinement statistics are reported in Table 1. The structure assembly was analyzed using PISA^82^.

### Sequence and structure analysis

Local alignments were performed with BLASTp^83^ v.2.12.0+ or DIAMOND^84^ v.2.0.15, and network analysis was carried out in Cytoscape^85^ v.3.9.1. Conserved domains at the sequence level were inferred from the Conserved Domain Database^86^ (CDD). Heuristic structural searches against the Protein Databank (PDB) were performed on the Dali server^87^. Structural visualization and alignments were performed with PyMOL^88^ v.2.0 and USFC Chimera^89^ v.1.16.

### Substrate docking

The BHET substrate was docked into the catalytic site of PET46 utilizing a combination of AutoDock3^90^ as a docking engine and DrugScore2018^91,92^ as an objective function. Following an established procedure^91,93^, the docking protocol considered 100 independent runs for BHET using an initial population size of 150 individuals, a maximum of 50,000 generations, a maximum of 10^6^ energy evaluations, a mutation rate of 0.02, a crossover rate of 0.8, and an elitism value of 1. The Lamarckian genetic algorithm was chosen for sampling in all approaches. Distances between atoms were measured using the PyMOL Molecular Graphics System^88^ v.2.3.0.

### PET degradation assays

Respectively, 3 µM PET46 WT (roughly 0.1 mg mL^−1^), the generated variants, IsPETase or LCC were incubated with 50 µM ethylene terephthalate linear trimer (3PET, Toronto Research Chemicals, Ontario, Canada), 150 µM bis-(2-hydroxyethyl) terephthalate (BHET), 150 µM mono-(2-hydroxyethyl) terephthalate (MHET; Merck, Darmstadt, Germany), 7 mg amorphous PET foil platelet (product no. ES301445; a = 5 mm^2^, 33.6 µmol or 168 mM TPA eq.; GoodFellow GmbH, Hamburg, Germany), 2 mg semi-crystalline PET powder (product no. ES306000; >40% crystallinity, d ≈ 300 µm; 9.6 µmol or 48 mM TPA eq.; GoodFellow GmbH, Hamburg, Germany), unless indicated otherwise. The reaction took place under agitation in 200 µL with 0.1 M potassium phosphate buffer pH 8 at 30, 50, 60, or 70 °C for a maximum of 5 days. Alternatively, 0.06 µM PET46 WT (roughly 2 µg mL^−1^), LCC or IsPETase were incubated with 150 mM BHET at 70, 50, or 30 °C, respectively, for up to 1 h. For end point analysis, samples were prepared in 96 well microtiter plates by adding 12.5 µL reaction supernatant to 50 µL acetonitrile with 1% v/v trifluoroacetic acid (TFA) followed by centrifugation (2,204 *g*, 30 min; A-2-DWP rotor, Eppendorf AG, Hamburg, Germany) and transferring of 50 µL centrifugation supernatant into 150 µL MilliQ H_2_O. Samples were sealed using ZoneFree™ sealing film (Excel Scientific, Victorville, CA, USA) and stored at −20 °C until analysis. Samples were analyzed via RP-UHPLC (UltiMate™ 3000 UHPLC system, Thermo Scientific, Waltham, MA, USA) with a Triart C18 column (YMC Europe GmbH, Dinslaken, Germany). Isocratic elution was done by using a mobile phase consisting of 20:80 (v/v) acetonitrile and water (acidified with 0.1% v/v trifluoroacetic acid) at a flowrate of 0.4 mL min^−1^^29,94^. Standards of the expected degradation products TPA, MHET, and BHET were analyzed to obtain the respective elution times. All assays were performed in triplicates and compared to an enzyme-free control.

### Biochemical characterization

Initial biochemical characterization aimed to identify the WT enzyme’s optimal temperature, pH, and substrate chain length and was performed with *para*-nitrophenyl (*p*NP) esters as described previously^25,29,94^. Shortly, 3 µM enzyme were incubated with 1 mM substrate in 0.1 M citrate, potassium phosphate, or carbonate-bicarbonate buffer and release of *p*NP-OH was measured at 405 nm on a BioTek Synergy H1 plate reader (Agilent Technologies Deutschland GmbH, Waldbronn, Germany). To test the thermostability of the enzyme, it was incubated at 60 and 70 °C for up to two weeks prior to a *p*NP assay under optimal conditions to quantify residual activity. Furthermore, the effect of metal ions, detergents, and organic solvents was assayed. The enzyme was either pre-incubated for one hour with 1 or 10 mM Ca^2+^, Co^2+^, Cu^2+^, Fe^3+^, Mg^2+^, Mn^2+^, Ni^2+^ or Zn^2+^ (chloride salts) or different detergents and organic solvents were added to the standard reaction mixture (Supplementary Fig. 10).

To test for general ferulic acid esterase activity, a colorimetric pH-shift-based assay with the model substrate ethyl cinnamate (EC) was performed as described previously^52^. In short, the reactions took place in 5 mM EPPS buffer with 0.45 mM phenol red. The release of protons due to enzymatic cleavage of the ester results in a decrease in absorbance at 550 nm, which is measured photometrically.

All assays were performed with 3 µM enzyme, unless otherwise indicated, in triplicates, and compared to an enzyme- or additive-free control.

#### Measuring of protein thermal stability

Approximately 1 mg mL^−1^ purified enzyme solution in 100 mM potassium phosphate buffer with 100 mM NaCl (pH 7.2) was loaded into Nano Temper capillary tubes. The capillary tubes were then loaded into a Prometheus nanoDSF (NanoTemper Technologies GmbH, Munich, Germany) and a melting scan with a range from 20 to 95 °C with a heating rate of 1 °C min^−1^ was performed. The results were evaluated with the NanoTemper analytics software.

### Reporting summary

Further information on research design is available in the Nature Portfolio Reporting Summary linked to this article.

### Supplementary information

Supplemental Information

Description of Additional Supplementary Files

Supplementary Data 1

Supplementary Data 2

Reporting Summary

## Data Availability

The crystal structure of PET46 is deposited in the PDB with accession 8B4U and provided in Supplementary Data 1. All data generated or analyzed during this study are included in this published article and its supplementary information files, including Supplementary Data 2. The corresponding author can provide further data that support the findings of this study upon reasonable request.
