# Supplementary material for: An archaeal lid-containing feruloyl esterase degrades polyethylene terephthalate
Source: Commun Chem. 2023 Sep 11;6:193. doi: 10.1038/s42004-023-00998-z (PMC10495362; doi:10.1038/s42004-023-00998-z)
Supplement: Supplementary file 1 — Supplemental Information [file 42004_2023_998_MOESM1_ESM.pdf]

An archaeal lid-containing feruloyl esterase degrades polyethylene terephthalate

Pablo Perez-Garcia[1,2,‡], Jennifer Chow[1,‡], Elisa Costanzi[3], Marno Gurschke[1], Jonas Dittrich[4], Robert F. Dierkes[1], Rebecka Molitor[5], Violetta Applegate[3], Golo Feuerriegel[1], Prince Tete[1], Dominik Danso[1], Stephan Thies[5], Julia Schumacher[3], Christopher Pfleger[4], Karl-Erich Jaeger[5,6], Holger Gohlke[4,7], Sander H. J. Smits[3,8], Ruth A. Schmitz[2,*], Wolfgang R. Streit[1,*]

Supplementary Figures

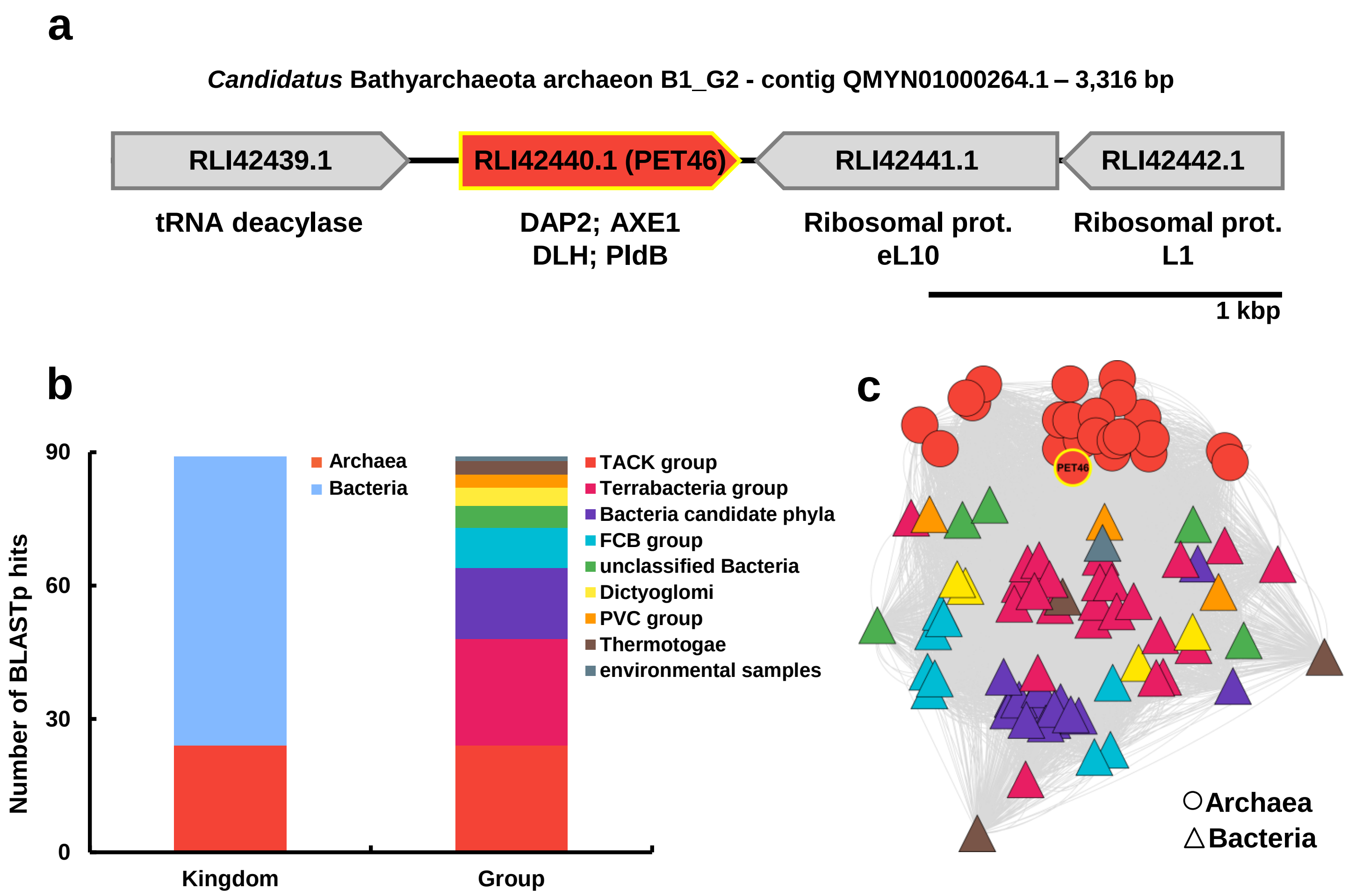

Supplementary Figure 1: Genetic context of *PET46* and protein homologs. PET46 is located in a small contig between genes coding for translation-associated proteins. It contains conserved sequence domains from dipeptidyl aminopeptidase/acylaminoacyl peptidase (DAP2), acetyl xylan esterase (AXE1), dienelactone hydrolase (DLH) or lysophospholipase (PldB, **a**). Archaeal homologs from PET46 derive mainly from other Bathyarchaeota, but there are more bacterial homologs (query cov. > 80%, seq. id. > 40%; **b**). A sequence network analysis displaying sequence identity reveals that PET46 and its archaeal homologs share high homology to sequences derived from Firmicutes/Bacillota (Terrabacteria) and others (**c**). Archaeal sequences are displayed as circles and bacterial as triangles. PET46 is highlighted with a yellow border. Color legend in “c” is shared with “b”.

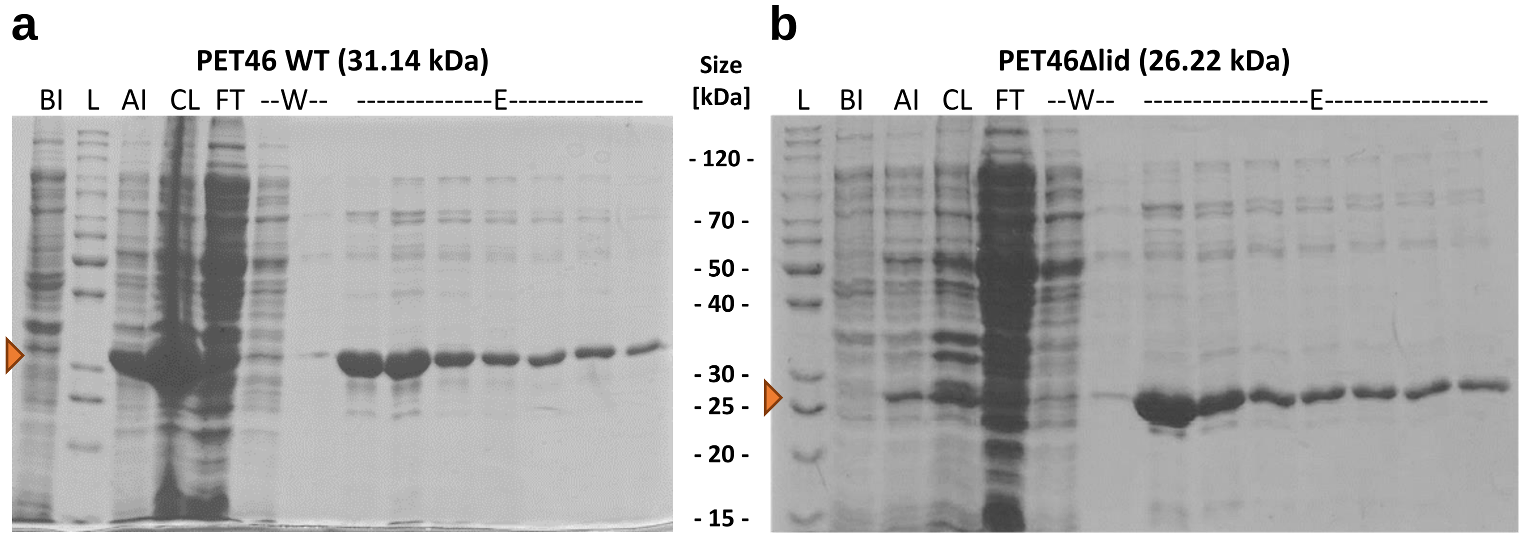

Supplementary Figure 2: Production and purification of PET46 wildtype (WT, a) and PET46Δlid (b). SDS-PAGE gels showing the protein production in *E. coli* BL21 (DE3) carrying the pET21a(+) plasmid with the corresponding gene and purification via immobilized metal affinity chromatography (IMAC). L: PageRuler™ Unstained Protein Ladder (26614, Thermo Fisher Scientific, Darmstadt, Germany); BI: before induction; AI: after induction; CL: cell lysate; FT: flow-through; W: washing steps; E: elution steps. The expected size of the protein of interest is pointed with a coral orange triangle.

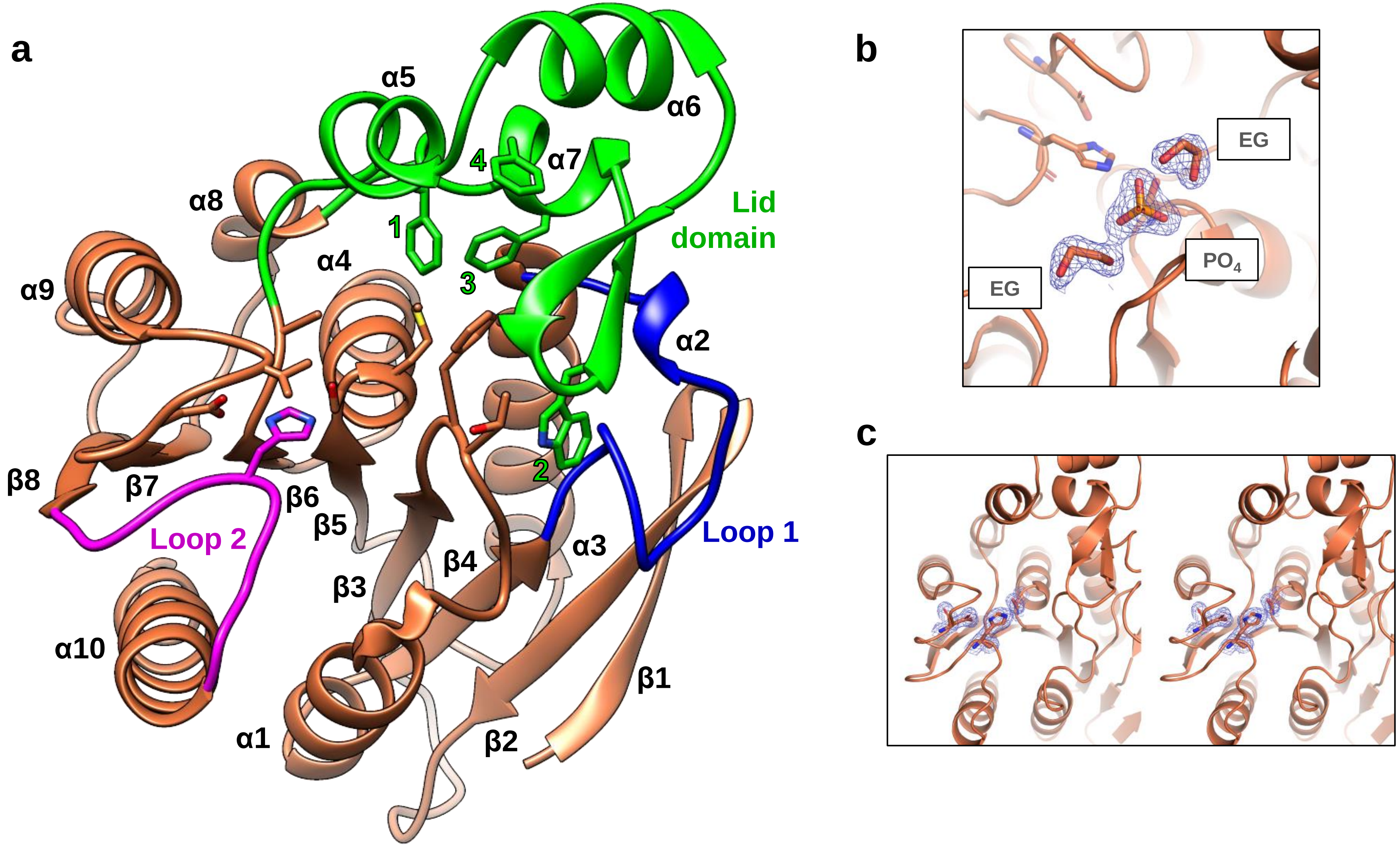

Supplementary Figure 3: The crystal structure of PET46 consists of 7 α-helixes and 8 β-strands forming the canonical α/β-hydrolase fold and 3 α-helixes and 2 anti-parallel β-strands making the lid. Together with the lid domain (bright green), Loop 1 and Loop 2 (deep blue and magenta) are the main structural variations with the IsPETase (**a**). These loops are conserved in all ferulic acid esterases (FAEs) analyzed. Displayed are the catalytic triad (see Figure 2 for the specific positions) and homologous residues involved in substrate binding in PETases or FAEs. The lid domain contains four aromatic residues towards the active site (Phe148, Trp172, Phe178, and Phe179; 1-4 bright green). 2Fo-Fc map contoured at one sigma is shown as blue mesh around the PO_4 and ethylene glycol (EG) moieties modelled near the active site (**b**). Stereo image of the density of the active site residues (**c**). The catalytic triad residues are shown as sticks.

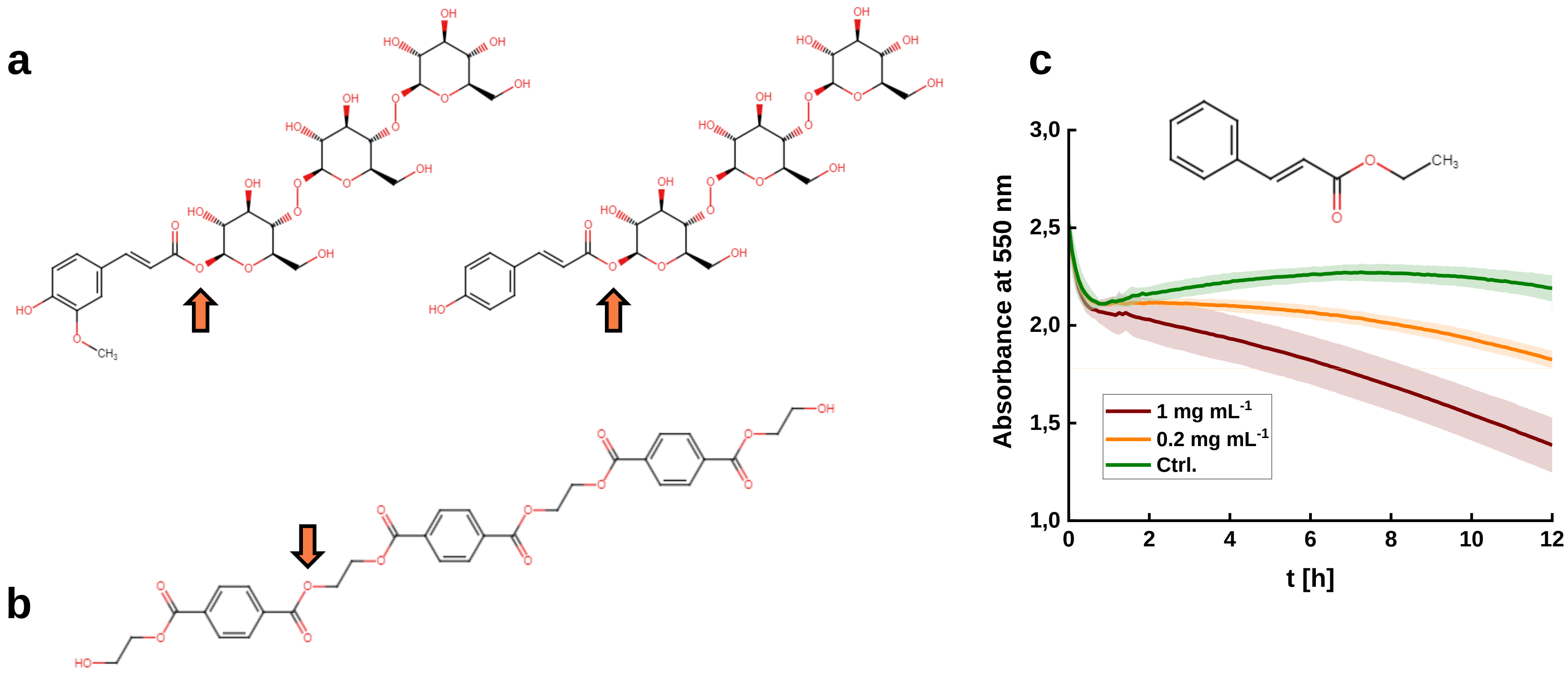

Supplementary Figure 4: Hydroxycinnamic acid-esters, the native substrates of ferulic acid esterases, are similar to the terminus of a PET polymer. A feruloyl-polysaccharide (left) and a *p*-coumaryl-polysaccharide (right) are two examples of hydroxycinnamic acid-polysaccharide esters (**a**). The synthetic ethylene terephthalate linear trimer (3PET) used as a substrate in this study (**b**). The attacked oxygen during an exo-reaction is highlighted with an arrow. PET46 degrades ethyl cinnamate (EC), a model substrate for FAE activity (**c**). A pH-shift assay (phenol red) with ethyl cinnamate (EC) and PET46 at two concentrations results in the release of H^+ upon ester hydrolysis.

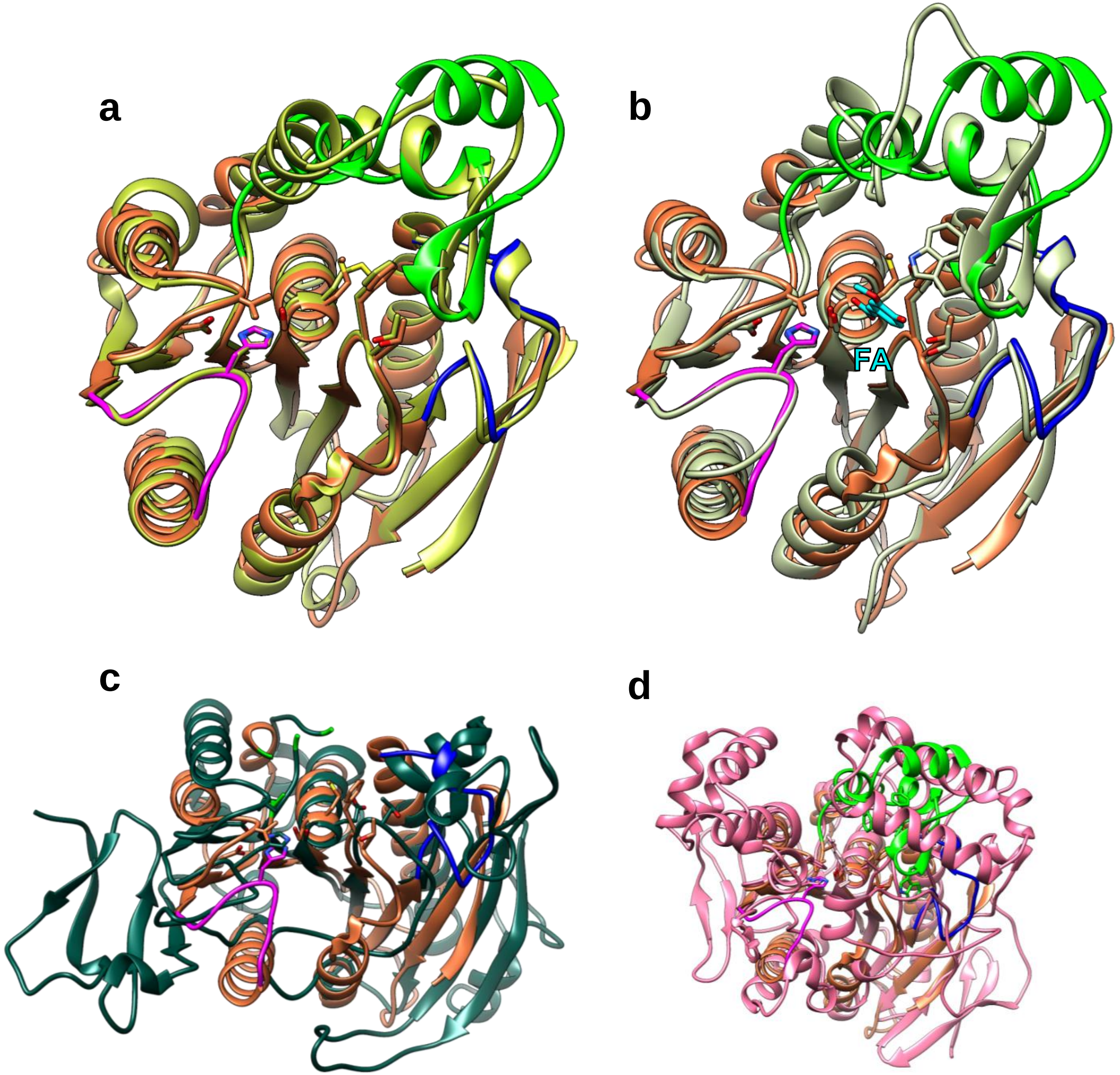

Supplementary Figure 5: The archaeal PETase PET46 is structurally homolog to ferulic acid esterases (FAEs). The crystal structure of PET46 (coral orange) is compared to the crystal structures of GthFAE from *Geobacillus thermoglucosidasius* (lime green; PDB 7WWH; **a**), the Est1E FAE from *Butyrivibrio proteoclasticus* (cream white; PDB 2WTN_A) bound to ferulic acid (FA; cyan; **b**), and the tannase IsMHETase from *I. sakaiensis* (petrol green; PDB 6QZ4; **c**). The lid domains of PET46 and IsMHETase have been omitted in "c" for better visualization (bright green). On the contrary, PET46 differs from the esterase TfCa from *Thermobifida fusca* (hot pink; PDB 7VVE) bound to MHETA (pink), but has the same mechanism of degrading 3PET[46] (**d**).

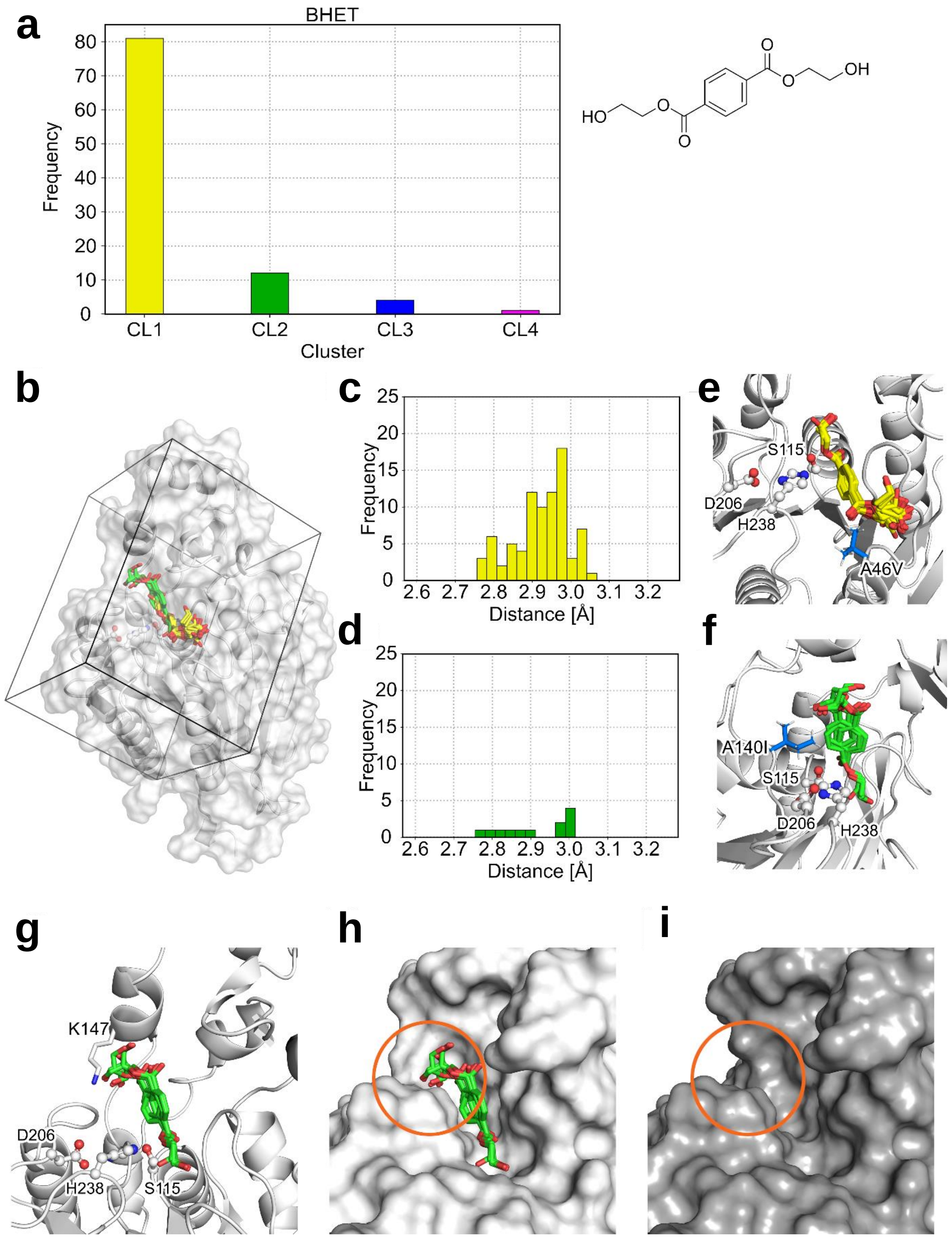

Supplementary Figure 6: Docking of BHET into PET46. Docking of BHET yielded four possible binding poses (clusters CL1-CL4) in PET46 (**a**). Docked poses of the two largest clusters within PET46 with the box depicting the search space (**b**). Distributions of the smallest distances between the docked substrate's carbonyl carbon and the hydroxyl oxygen from the catalytic serine for the two largest clusters (**c**, **d**). Location of the substituted amino acids in the A46V variant (blue sticks, **e**), the A140I variant (blue sticks, **f**), and the Lys147 variant (white sticks, **g**) of PET46. The comparison of the substrate binding sites for the WT (white surface, **h**) and the K147A variant (gray surface, **i**) shows an extended substrate binding site in the variant (orange circles). The same orientation is used like for "g".

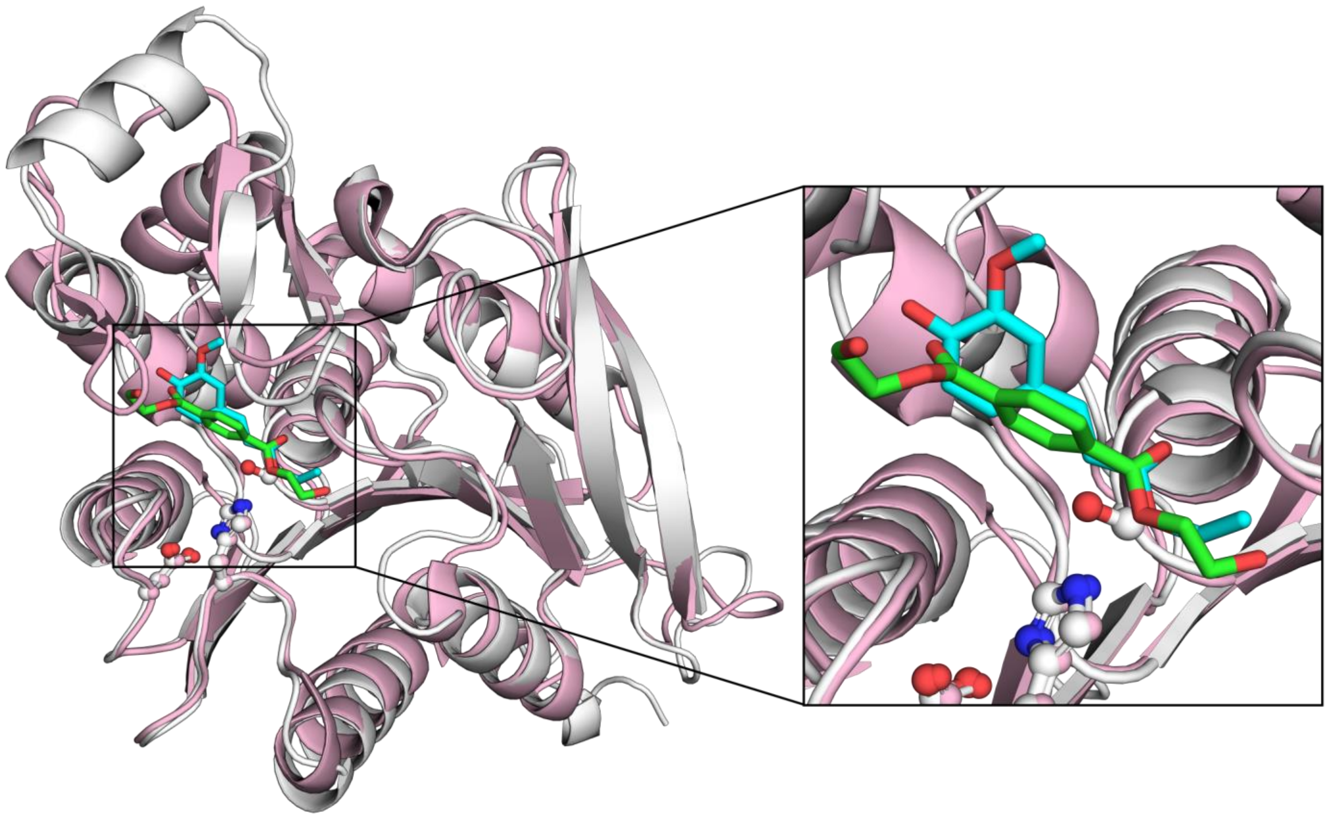

Supplementary Figure 7: Comparison of potential binding poses of BHET within PET46 with the binding pose of ethylferulate in the crystal structure of the cinnamoyl esterase LJ0536 S106A variant. An overlay of both protein structures, i.e., PET46 (grey) and the cinnamoyl esterase LJ0536 S106A variant (pink, PDB 3QM1), including their substrates, i.e., the docked BHET (green) and crystalized ethylferulate (cyan), reveals that the carbonyl moiety is positioned virtually identically within the binding pocket.

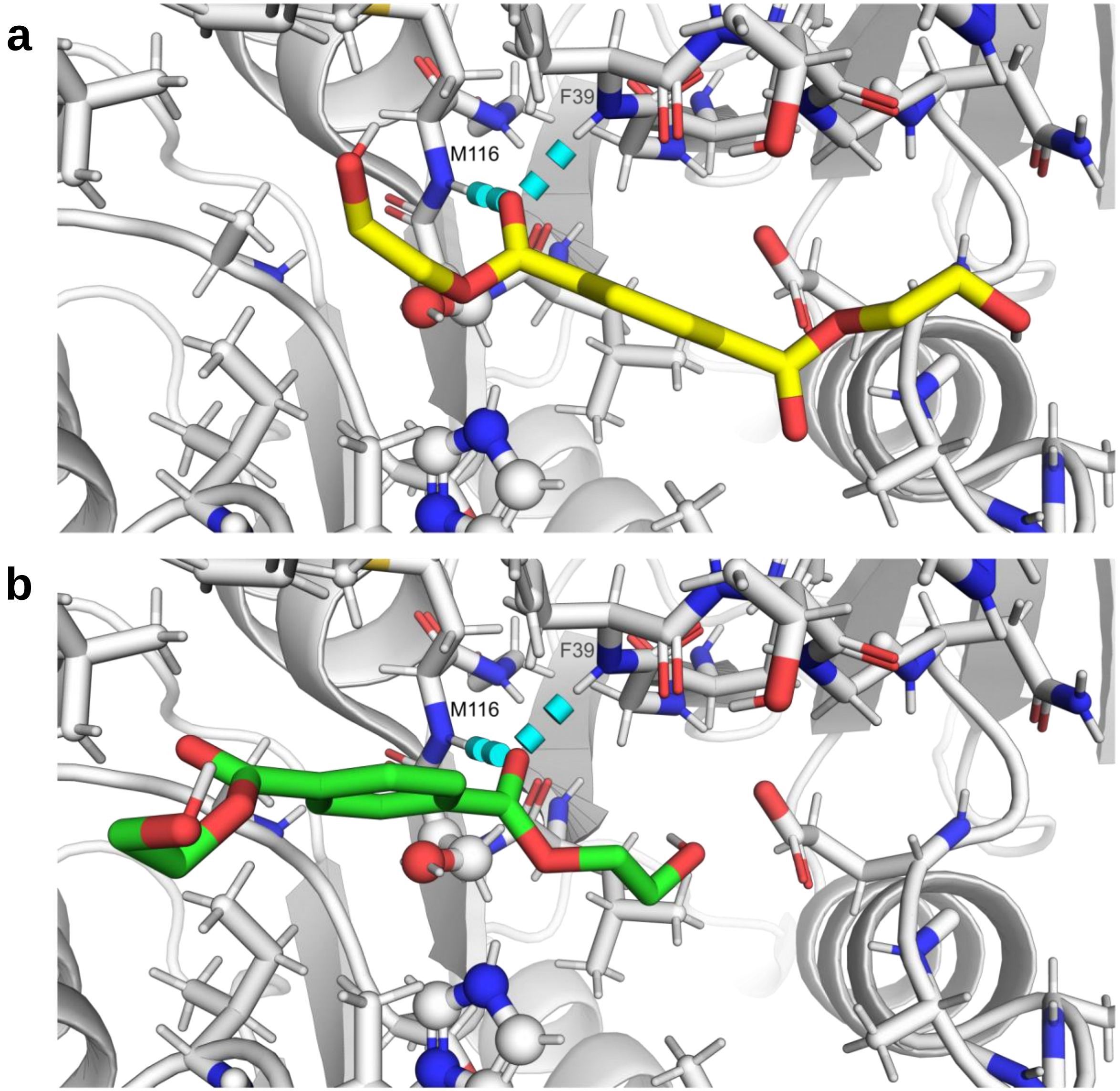

Supplementary Figure 8: Oxyanion hole stabilizes carbonyl oxygen of BHET. For the binding poses of BHET within the two largest clusters (CL1 and CL2), the backbone amides of Phe39 and Met116 form an oxyanion hole. For the binding pose of CL1 (**a**) and CL2 (**b**), we find distances (cyan cylinders) between the amide hydrogens and the carbonyl oxygen between 1.9 and 2.2 Å.

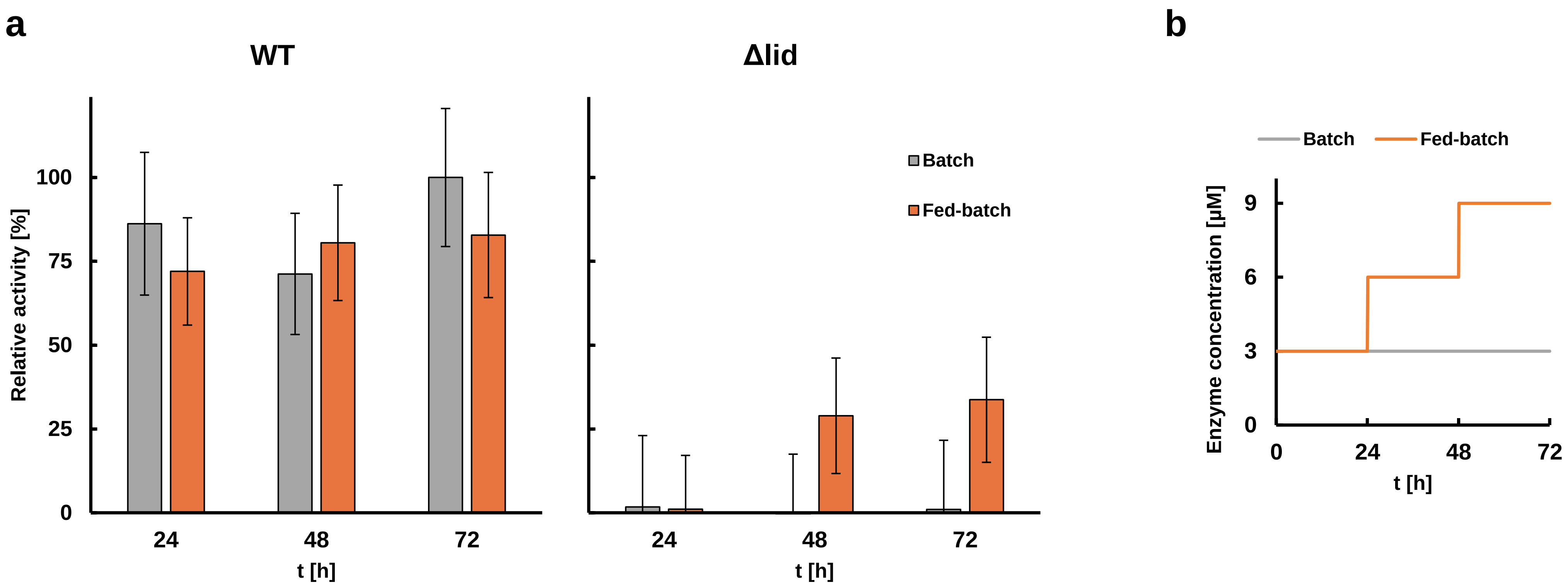

Supplementary Figure 9: Batch vs. fed-batch PET degradation by PET46 WT and Δlid. Experiments show that the lid domain is crucial for activity on the polymer. No significant differences were observed between batch and fed-batch process for the WT, indicating that degradation occurs mostly in the first 24 h and does not increase by addition of fresh enzyme. Therefore, activity might correspond solely to cleavage of terminal aromatic moieties. In the case of the lidless variant (Δlid), virtually no activity was measured in batch mode and only 33.72% relative activity was measured after 3 days (**a**). The experimental set-up was as in Figure 5a, where 3 µM PET46 were incubated with 2 mg semi-crystalline PET powder (9.6 µmol or 48 mM TPA eq.) at 60 °C for a total of 72 h. In this case, we sampled the reaction every 24 h and compared to a parallel reaction in which we added 3 µM fresh PET46 every 24 h after sampling (**b**). The same amount of buffer was added to the batch reaction to account for the dilution factor. Activities are shown relative to the maximum amount of product released by PET46 WT after 72 h in batch mode (1.6 mM; Figure 5a). Error bars indicate the standard deviation (n=3).

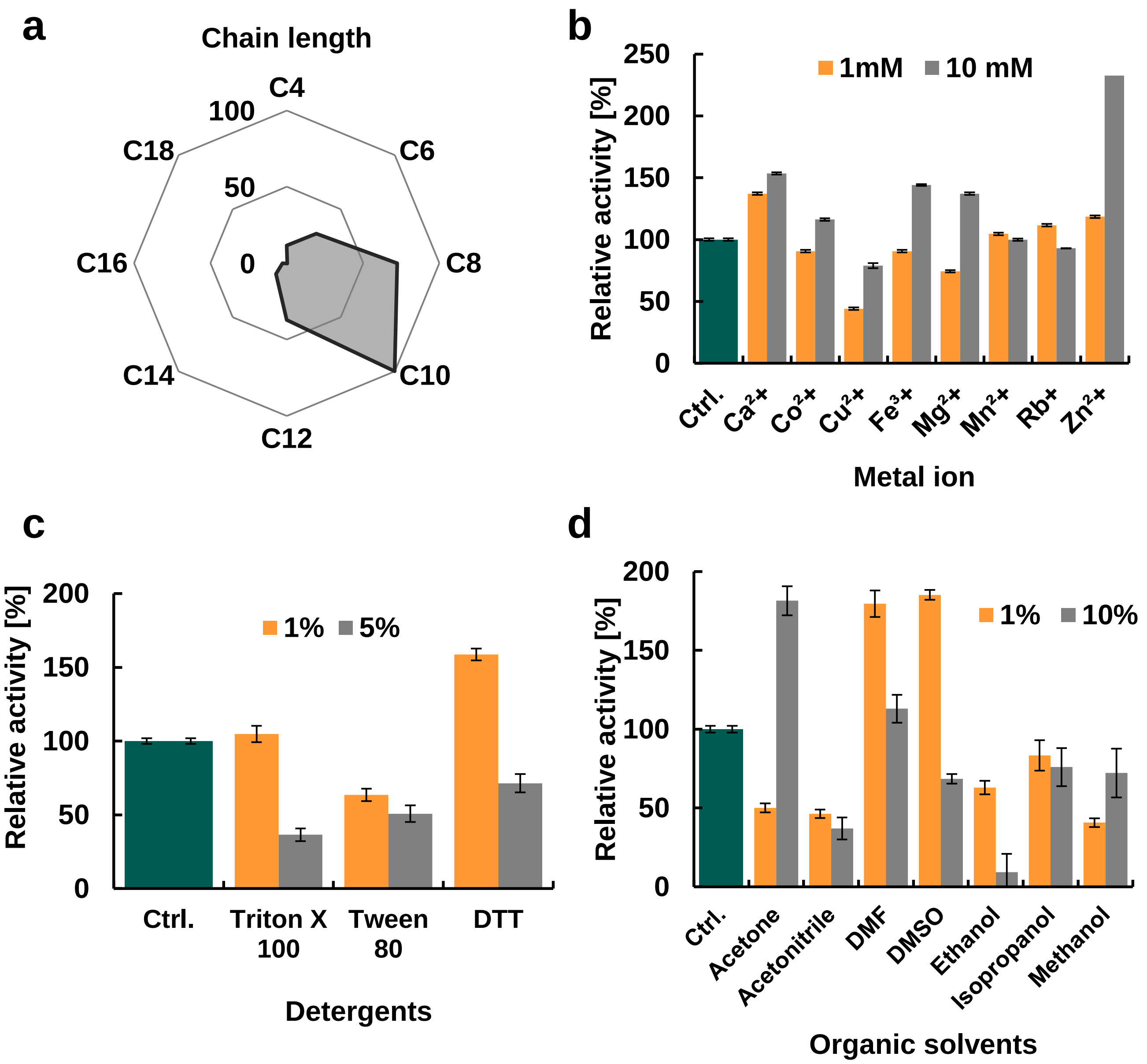

Supplementary Figure 10: Biochemical characterization of PET46. Optimal *p*NP-ester acyl chain length was determined (**a**). The effect of metal ions (**b**), detergents (**c**) and organic solvents (**d**) on the activity of PET46 was studied compared to an additive-free control (Ctrl.). Error bars indicate the standard deviation (n=3). Standard deviation in “a“ was below 6%.

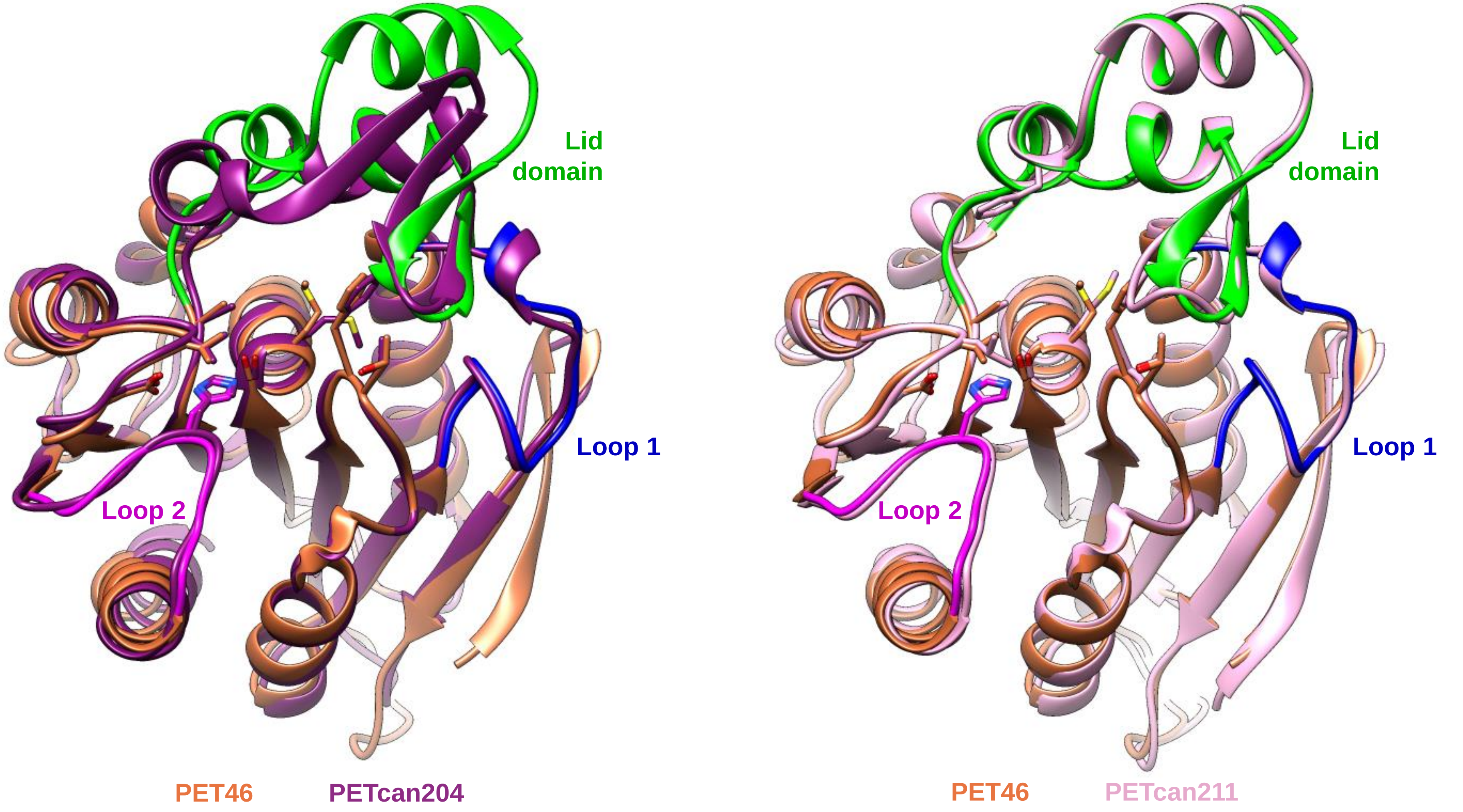

Supplementary Figure 11: PET46 vs. archaeal PET-degrading PETcan204 and PETcan211[60]. PETase PETcan204 is 97.93% identical to WP_187147021.1 and WP_148684612.1 from *Thermophilum adornatum*, while PETcan211 is 99.62% identical to MBS7639053.1 from a Cand. Bathyarchaeota archaeon, both archaea. While PET46 has a sequence identity of 34.92% and 65.78% to the other enzymes, respectively, their structures are very similar. Loop 1, Loop 2 (deep blue and magenta in PET46), and all the key residues shown are conserved, and they both contain a lid domain (bright green in PET46). While the lid of PETcan211 is very similar to PET46′s, PETcan204 contains a third β-sheet instead of PET46′s α6 (top, Supplementary Fig S3). PETcan204 and PETcan211 structures are AlphaFold models retrieved from the original publication.

An archaeal lid-containing feruloyl esterase degrades polyethylene terephthalate

Pablo Perez-Garcia[1,2,‡], Jennifer Chow[1,‡], Elisa Costanzi[3], Marno Gurschke[1], Jonas Dittrich[4], Robert F. Dierkes[1], Rebecka Molitor[5], Violetta Applegate[3], Golo Feuerriegel[1], Prince Tete[1], Dominik Danso[1], Stephan Thies[5], Julia Schumacher[3], Christopher Pfleger[4], Karl-Erich Jaeger[5,6], Holger Gohlke[4,7], Sander H. J. Smits[3,8], Ruth A. Schmitz[2,*], Wolfgang R. Streit[1,*]

SUPPLEMENTARY TABLES

Supplementary Table 1: Primers used in this study. Lid deletion and point mutations were introduced by site-directed mutagenesis. pET primers were used for Sanger sequencing to verify the correctness of the produced variants prior to expression.

**Primer Name**	**Nucleotide Sequence (5‘->3‘)**	**T_m [°C]**	**Reference**
Δlid_for	TTCTACCTTAGATGTGCTGGATCGC	67.1	This work
Δlid_rev	GAGTCCCATGCACTATACAGAATAACAAATTTAATG	67.3	This work
K147A_for	AGCCTTAACCCCGCTGCGCCGCGCGTTTCTG	81.9	This work
K147A_rev	GACGAATTGATTCTTTTTCTAAGCCTTCCAGAAACGCGCGGCG	77.7	This work
A46V_for	TTTCATGGCTTTACCGGCAATAAATCAGAAGTGCATCGTCTGT	76.0	This work
A46V_rev	GCGTGCAACATGAACAAACAGACGATGCACTTCTGATTT	75.2	This work
A140I_for	TCGTCGCATTAAATTTGTTATTCTGTATAGTGCAATTTTAACCCCGC	73.9	This work
A140I_rev	ATTTGCGGCGCAGCGGGGTTAAAATTGCACTATAC	75.9	This work
pET_for	ATATAGGCGCCAGCAACC	62.7	Novagen/Merck (Darmstadt, Germany)
pET_rev	TCCGGATATAGTTCCTC	54.3	Novagen/Merck (Darmstadt, Germany)
