# Supplementary material for: An archaeal lid-containing feruloyl esterase degrades polyethylene terephthalate
Source: Commun Chem. 2023 Sep 11;6:193. doi: 10.1038/s42004-023-00998-z (PMC10495362; doi:10.1038/s42004-023-00998-z)
Supplement: Supplementary file 2 — Description of Additional Supplementary Files [file 42004_2023_998_MOESM2_ESM.pdf]

Description of Additional Supplementary Files

File name: Supplementary Data 1

Description: structure of PET46 WT (PDB 8B4U).

File name: Supplementary Data 2

Description: numerical source data for graphs and charts.
