# Supplementary material for: An archaeal lid-containing feruloyl esterase degrades polyethylene terephthalate
Source: Commun Chem. 2023 Sep 11;6:193. doi: 10.1038/s42004-023-00998-z (PMC10495362; doi:10.1038/s42004-023-00998-z)
Supplement: Supplementary file 5 — Reporting Summary [file 42004_2023_998_MOESM5_ESM.pdf]

nature portfolio

Corresponding author(s): Prof. Dr. Ruth A. Schmitz and Prof. Dr. Wolfgang R. Streit

Last updated by author(s): Aug 10, 2023

Reporting Summary

Nature Portfolio wishes to improve the reproducibility of the work that we publish. This form provides structure for consistency and transparency in reporting. For further information on Nature Portfolio policies, see our Editorial Policies and the Editorial Policy Checklist.

Statistics

For all statistical analyses, confirm that the following items are present in the figure legend, table legend, main text, or Methods section.

n/a	Confirmed	
☐	☒	The exact sample size (*n*) for each experimental group/condition, given as a discrete number and unit of measurement
☐	☒	A statement on whether measurements were taken from distinct samples or whether the same sample was measured repeatedly
☐	☒	The statistical test(s) used AND whether they are one- or two-sided<br>*Only common tests should be described solely by name; describe more complex techniques in the Methods section.*
☒	☐	A description of all covariates tested
☒	☐	A description of any assumptions or corrections, such as tests of normality and adjustment for multiple comparisons
☐	☒	A full description of the statistical parameters including central tendency (e.g. means) or other basic estimates (e.g. regression coefficient) AND variation (e.g. standard deviation) or associated estimates of uncertainty (e.g. confidence intervals)
☒	☐	For null hypothesis testing, the test statistic (e.g. *F*, *t*, *r*) with confidence intervals, effect sizes, degrees of freedom and *P* value noted<br>*Give P values as exact values whenever suitable.*
☒	☐	For Bayesian analysis, information on the choice of priors and Markov chain Monte Carlo settings
☒	☐	For hierarchical and complex designs, identification of the appropriate level for tests and full reporting of outcomes
☒	☐	Estimates of effect sizes (e.g. Cohen's *d*, Pearson's *r*), indicating how they were calculated

Our web collection on statistics for biologists contains articles on many of the points above.

Software and code

Policy information about availability of computer code

Data collection	Biotek Gen5 v3.09 was used for collection of the protein activity measurements (pNP and phenol-red assays). Bruker HyStar v3.2 (build 44) was used to aquire UPLC data. NanoTemper analytics software was used for collection of nanoDSF experiments data.
Data analysis	Microsoft Excel and Bruker HyStar v.3.2 (build 44) were used for the analysis of activity measurements. The HMMER v.3.3 package was used for HMM searches. Diamond v.2.0.15 was used for all-against-all BLASTp searches. Network analysis was performed with Cytoscape v.3.9.1. Structural visualization and alignments were performed with PyMOL v.2.0/v.2.3 and USFC Chimera v.1.16. AutoDock3 and DrugScore2018 were used for substrate docking. The CCP4 Suite, XDS, Aimless, Phaser, AlphaFold, COOT, Phenix and PISA were used to solve the crystal structure of PET46. NanoTemper analytics software was used to analyze nanoDSF data and calculate Tm.

For manuscripts utilizing custom algorithms or software that are central to the research but not yet described in published literature, software must be made available to editors and reviewers. We strongly encourage code deposition in a community repository (e.g. GitHub). See the Nature Portfolio guidelines for submitting code & software for further information.

Data

Policy information about availability of data

All manuscripts must include a data availability statement. This statement should provide the following information, where applicable:

- Accession codes, unique identifiers, or web links for publicly available datasets
- A description of any restrictions on data availability
- For clinical datasets or third party data, please ensure that the statement adheres to our policy

The crystal structure of PET46 is deposited in the PDB with accession 8B4U and provided in Supplementary Data 1. All data generated or analyzed during this study are included in this published article and its supplementary information files, including Supplementary Data 2. The corresponding author can provide further data that support the findings of this study upon reasonable request.

Research involving human participants, their data, or biological material

Policy information about studies with human participants or human data. See also policy information about sex, gender (identity/presentation), and sexual orientation and race, ethnicity and racism.

Reporting on sex and gender	Our study does not involve human participants.
Reporting on race, ethnicity, or other socially relevant groupings	Our study does not involve human participants.
Population characteristics	Our study does not involve human participants.
Recruitment	Our study does not involve human participants.
Ethics oversight	Our study does not involve human participants.

Note that full information on the approval of the study protocol must also be provided in the manuscript.

Field-specific reporting

Please select the one below that is the best fit for your research. If you are not sure, read the appropriate sections before making your selection.

☒ Life sciences ☐ Behavioural & social sciences ☐ Ecological, evolutionary & environmental sciences

For a reference copy of the document with all sections, see nature.com/documents/nr-reporting-summary-flat.pdf

Life sciences study design

All studies must disclose on these points even when the disclosure is negative.

Sample size	For experiments concerning activity measurements, each measurement consisted of at least three replicates takes (n>3). Each experiment was repeated at least three times. As the standard deviations were low and internal controls consistent, this sample size was considered as sufficient.
Data exclusions	Data were not excluded from analysis.
Replication	All activity measurements were performed independently at least three times.
Randomization	Randomization does not apply to our study.
Blinding	Blinding does not apply to our study.

Reporting for specific materials, systems and methods

We require information from authors about some types of materials, experimental systems and methods used in many studies. Here, indicate whether each material, system or method listed is relevant to your study. If you are not sure if a list item applies to your research, read the appropriate section before selecting a response.

Materials & experimental systems

n/a	Involved in the study
☒	☐ Antibodies
☒	☐ Eukaryotic cell lines
☒	☐ Palaeontology and archaeology
☒	☐ Animals and other organisms
☒	☐ Clinical data
☒	☐ Dual use research of concern
☒	☐ Plants

Methods

n/a	Involved in the study
☒	☐ ChIP-seq
☒	☐ Flow cytometry
☒	☐ MRI-based neuroimaging
